# Target Molecules of STIM Proteins in the Central Nervous System

**DOI:** 10.3389/fnmol.2020.617422

**Published:** 2020-12-23

**Authors:** Karolina Serwach, Joanna Gruszczynska-Biegala

**Affiliations:** Molecular Biology Unit, Mossakowski Medical Research Centre, Polish Academy of Sciences, Warsaw, Poland

**Keywords:** STIM, SOCE components, glutamate receptors, Ca^2+^ channels, calcium signaling, STIM regulators and effectors, store-operated calcium entry (SOCE), central nervous system

## Abstract

Stromal interaction molecules (STIMs), including STIM1 and STIM2, are single-pass transmembrane proteins that are located predominantly in the endoplasmic reticulum (ER). They serve as calcium ion (Ca^2+^) sensors within the ER. In the central nervous system (CNS), they are involved mainly in Orai-mediated store-operated Ca^2+^ entry (SOCE). The key molecular components of the SOCE pathway are well-characterized, but the molecular mechanisms that underlie the regulation of this pathway need further investigation. Numerous intracellular target proteins that are located in the plasma membrane, ER, cytoskeleton, and cytoplasm have been reported to play essential roles in concert with STIMs, such as conformational changes in STIMs, their translocation, the stabilization of their interactions with Orai, and the activation of other channels. The present review focuses on numerous regulators, such as Homer, SOCE-associated regulatory factor (SARAF), septin, synaptopodin, golli proteins, partner of STIM1 (POST), and transcription factors and proteasome inhibitors that regulate STIM-Orai interactions in the CNS. Further we describe novel roles of STIMs in mediating Ca^2+^ influx via other than Orai pathways, including TRPC channels, VGCCs, AMPA and NMDA receptors, and group I metabotropic glutamate receptors. This review also summarizes recent findings on additional molecular targets of STIM proteins including SERCA, IP_3_Rs, end-binding proteins (EB), presenilin, and CaMKII. Dysregulation of the SOCE-associated toolkit, including STIMs, contributes to the development of neurodegenerative disorders (e.g., Alzheimer's disease, Parkinson's disease, and Huntington's disease), traumatic brain injury, epilepsy, and stroke. Emerging evidence points to the role of STIM proteins and several of their molecular effectors and regulators in neuronal and glial physiology and pathology, suggesting their potential application for future therapeutic strategies.

## Introduction

Calcium ion (Ca^2+^) is a second messenger of crucial importance to neurons as it participates in the transmission of the depolarizing signals and contributes to synaptic activity and apoptosis. Cytoplasmic Ca^2+^ level in neurons is regulated in a comprehensive way via the components localized in the plasma membrane (PM) such as ion channels, exchangers, and pumps, as well as the components localized in the mitochondria, endoplasmic reticulum (ER), Golgi apparatus, and nucleus (Brini et al., 2014).

Plasma membrane Ca^2+^ channels in neurons are divided into three major groups according to their mechanism of action: voltage-gated Ca^2+^ channels (VGCC), receptor-operated Ca^2+^ channels (ROC: α-amino-3-hydroxy-5-methyl-4-isoxazole propionic acid receptors [AMPARs] and *N*-methyl-D-aspartate receptors [NMDARs]), and store-operated channels (SOC; Orai, transient receptor potential cation [TRPC] channels, arachidonate-regulated Ca^2+^ [ARC] channels) (Brini et al., 2014). In neurons, Ca^2+^ entry from the extracellular space is mediated via VGCCs and glutamate receptors (ionotropic: AMPAR, NMDAR, and metabotropic: mGluR) and is complemented by store-operated Ca^2+^ entry (SOCE) (Brini et al., 2014). Noteworthy, SOCE is the main Ca^2+^ source in resting neurons, while after depolarization Ca^2+^ influx is mediated mainly via VGCC, NMDAR and AMPAR (Brini et al., 2014). mGluR mediates both rapid transient depolarization and prolonged depolarization (Brini et al., 2014). Two systems responsible for Ca^2+^ extrusion from the cytoplasm to the extracellular milieu are PM Ca^2+^ adenosine triphosphatase (PMCA) and PM Na^+^/Ca^2+^ exchanger (NCX). While NCX affinity to Ca^2+^ is low, its capacity is high. Contrary, PMCA is characterized by opposite properties (Blaustein et al., 2002; Brini and Carafoli, 2011; Brini et al., 2014).

Mitochondria are also essential components of neuronal Ca^2+^ toolkit. They modulate intensity and duration of Ca^2+^ signals following extracellular stimuli (Duszyński et al., 2006). Since they have the ability to accumulate Ca^2+^, they function as Ca^2+^ buffers. Mitochondria localized in close proximity to Ca^2+^ channels are exposed to high Ca^2+^ level and can accumulate Ca^2+^ efficiently. This decreases local Ca^2+^ level and results in depletion of ER Ca^2+^ stores and activation of SOCE (Duszyński et al., 2006; Spät and Szanda, 2017). Special communication between mitochondria and the ER also enables Ca^2+^ release from the ER to mitochondria and its accumulation in the mitochondrial matrix. Increased Ca^2+^ concentration in the mitochondrial matrix stimulates the energy metabolism and boosts the activity of the tricarboxylic acid cycle enzymes, providing reducing equivalents to the respiratory chain and thus influencing the production of ATP (Brini et al., 2014). Calcium influx during SOCE results in mitochondrial Ca^2+^ uptake, which in turn boosts mitochondrial energy metabolism. If Ca^2+^ overload appears, it may cause cell apoptosis (Spät and Szanda, 2017). Thus, mitochondria link cell metabolism with Ca^2+^ signaling and homeostasis (Duszyński et al., 2006).

Neuronal Ca^2+^ signaling also appears to be pivotal in the nucleus. Cell depolarization propagates Ca^2+^ to the nucleus where they target the CREB transcription factor and DREAM transcriptional repressor, thereby affecting the transcription of many genes (Dick and Bading, 2010).

In neurons, the ER constitutes a vital Ca^2+^ storage organelle. Release of Ca^2+^ from the ER occurs via ryanodine receptor (RyR) and inositol-1,4,5-trisphosphate 3 (IP_3_) receptor (IP_3_R). Ca^2+^ release through IP_3_R occurs in response to mGluR activation in the PM. In turn, an elevated level of cytoplasmic Ca^2+^ is the major trigger for Ca^2+^ release via RyR in the mechanism known as Ca^2+^-induced Ca^2+^ release (CICR) (Brini et al., 2014). The decreased Ca^2+^ level in the ER is refilled by SOCE.

SOCE is based on the influx of Ca^2+^ from the extracellular environment through channels of the PM and the replenishment of these ions in the ER when their levels decrease because of release into the cytoplasm (Blaustein and Golovina, 2001; Putney, 2003). The depletion of ER Ca^2+^ stores is detected by stromal interaction molecules (STIMs), including STIM1 and STIM2 proteins, that are sensors of Ca^2+^ levels in the ER (Liou et al., 2005; Roos et al., 2005; Zhang et al., 2005). After the activation of IP_3_Rs, the drop in Ca^2+^ concentration in the ER (Berridge et al., 2000) causes the oligomerization of STIM proteins and their movement toward ER-PM junctions (Liou et al., 2005; Zhang et al., 2005; Wu et al., 2006; Serwach and Gruszczynska-Biegala, 2019). At these junctions, STIM proteins form complexes with proteins of Ca^2+^ release-activated channels (CRACs) that are formed by Orais or SOCs that consist of Orais and TRPC channels, leading to the activation of these channels (Liou et al., 2005; Mercer et al., 2006; Soboloff et al., 2006c; Liao et al., 2008; Salido et al., 2009; Saul et al., 2014; Albarran et al., 2016). Two types of Ca^2+^ currents are caused by Ca^2+^ store depletion: I_CRAC_ (mediated by the activation of Orai1 and STIM1) and I_SOC_ (involving Orai1, TRPC1, and STIM1; Desai et al., 2015). Channel activation results in Ca^2+^ influx from the extracellular milieu to the cytoplasm (Prakriya et al., 2006), and then Ca^2+^ is taken to the ER by sarco/endoplasmic reticulum Ca^2+^-adenosine triphosphatase (SERCA) pump.

The interaction between STIM proteins and Orai1 is widely known to be essential for the proper function of SOCE in non-excitable cells. SOCE is a ubiquitous cell signaling pathway that is also present in many other tissues, including the rodent and human brain (Moccia et al., 2015) where it is involved in the regulation of intracellular ionic equilibrium and determines the excitability of neurons (Emptage et al., 2001; Gemes et al., 2011; Sun et al., 2014; Majewski and Kuznicki, 2015).

STIM proteins were originally described in non-excitable cells. They are now known to be present in most cells, including excitable cells, such as neurons, where STIM2 protein is predominantly expressed (Berna-Erro et al., 2009; Skibinska-Kijek et al., 2009; Gruszczynska-Biegala et al., 2011; Steinbeck et al., 2011). The primary function of STIM2 in neurons was suggested to be the regulation of resting levels of Ca^2+^ in the ER and Ca^2+^ leakage (Gruszczynska-Biegala et al., 2011; Gruszczynska-Biegala and Kuznicki, 2013). The main function of STIM1 in neurons appears to involve the activation of SOCE (Gruszczynska-Biegala et al., 2011). Various studies have also identified STIM proteins in neuroglial cells, such as astroglia, tumor cells of astroglial origin, oligodendrocyte progenitor cells (OPCs), and microglia (Kettenmann and Bruce, 2013; Verkhratsky and Parpura, 2014; Kraft, 2015; Molnár et al., 2016). Although both STIM1 and STIM2 are expressed in astroglia, STIM1 is thought to be the more abundant isoform in these cells (Gruszczynska-Biegala et al., 2011; Kraft, 2015). Gruszczynska-Biegala et al. showed that *Stim1* mRNA levels in both astroglial and neuronal cortical cultures were similar (Gruszczynska-Biegala et al., 2011).

In addition to interactions with and gating Orai, STIMs were found to recognize numerous interaction partners other than Orai. Thus, the present review focuses on the most important highly divergent target molecules of STIM proteins including positive and negative effectors and regulators in the central nervous system (CNS), mainly in neurons and glia. Recent data revealed a key role for STIM in several physiological and pathological conditions, including hypoxic/ischemic neuronal injury, traumatic brain injury (TBI), epilepsy, Alzheimer's disease (AD), Parkinson's disease (PD), and Huntington's disease (HD; Berna-Erro et al., 2009; Gemes et al., 2011; Steinbeck et al., 2011; Sun et al., 2014; Zhang et al., 2014, 2015; Popugaeva et al., 2015; Rao et al., 2015; Vigont et al., 2015; Tong et al., 2016; Czeredys et al., 2018; Serwach and Gruszczynska-Biegala, 2019). Therefore, studies of SOCE and STIM proteins may elucidate pathogenic mechanisms that are involved in the development of these diseases. Consequently, positive and negative modulators of STIM protein function or translocation may have many potential therapeutic applications. Thus, we also briefly discuss the pathophysiological significance of STIM protein interactions with their target proteins.

## STIM Protein Structure

**Figure 1:**
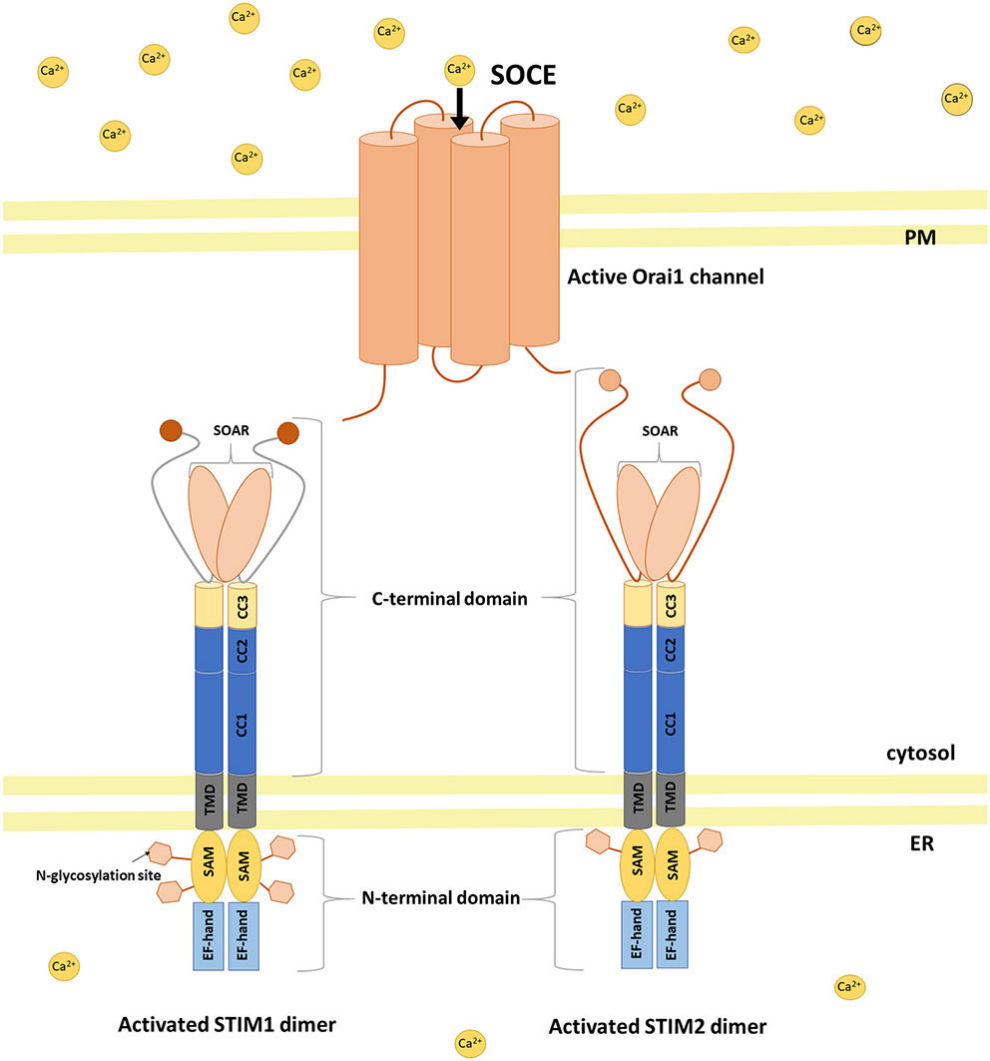
STIM activation and coupling with Orai1 in a mechanism of SOCE. Activation of STIM dimers is initiated by low Ca^2+^ concentration in the ER. STIMs oligomerize and migrate to ER–PM junctions where they activate SOC channels (e.g., Orai1) causing Ca^2+^ influx from the extracellular milieu to the cytoplasm and then refilling ER Ca^2+^ stores. SAM sterile alpha motif, TMD transmembrane domain, CC coiled-coil, SOAR STIM–Orai-activating region.

STIM1 and STIM2 proteins are encoded by the *STIM1* and *STIM2* genes, respectively, in humans (Williams et al., 2001). They are type 1 transmembrane proteins that are localized in the ER, although STIM1 was also found in the PM (Williams et al., 2001; Liou et al., 2005; Roos et al., 2005; Keil et al., 2010). Both isoforms contain luminal and cytosolic domains (Figure 1; Soboloff et al., 2012; Moccia et al., 2015). The ER luminal N-terminal domain consists of a conserved cysteine pair, a Ca^2+^-binding canonical EF-hand (cEF) domain, a non-Ca^2+^-binding hidden EF-hand (hEF) domain, a sterile α-motif (SAM) with one (for STIM2) or two (for STIM1) N-glycosylation sites, and a transmembrane domain (TMD). The cytosolic C-terminus includes three coiled-coil regions (CC1, CC2, and CC3) with a STIM–Orai-activating region (SOAR). The SOAR contains four α-helices (Sα1, Sα2, Sα3, and Sα4) and a KIKKKR sequence, which is required for the activation of Orai1 (Yuan et al., 2009; Yang et al., 2012). The CRAC activation domain (CAD) and Orai1-activating small fragment (OASF) are both larger than the SOAR, contain a CC1 region, and activate Orai1 (Muik et al., 2009; Park et al., 2009). SOAR function is inhibited by an inhibitory helix that is localized in Cα3 (Yang et al., 2012). Downstream of SOAR is an acidic inhibitory domain (ID) that also mediates the fast Ca^2+^-dependent inactivation of Orai1 (Lee et al., 2009). The C-terminus tail of STIM proteins also contains a proline/serine-rich (PS) domain, a microtubule-interacting domain, and a polybasic lysine-rich domain that is responsible for phospholipid interaction in the PM (Soboloff et al., 2012).

STIM1 and STIM2 proteins diverge significantly within the C-terminus (Figure 1). Strong evidence indicates that STIM proteins associate *in vivo*, and these interactions may be mediated by an association between CC regions of C-terminal ends of these proteins (Soboloff et al., 2006a). Notably, STIM2 has lower affinity for Ca^2+^ sensing compared with STIM1 because of three amino acid substitutions in cEF that allow STIM2 to detect smaller decreases in Ca^2+^ inside the ER lumen (Brandman et al., 2007; Hoth and Niemeyer, 2013).

## STIM Proteins in the Physiology of Neurons, Autophagy, and Neurodegenerative Diseases

### Physiology

By activating neuronal SOCE, STIM proteins play a pivotal role in the physiology of neurons (Serwach and Gruszczynska-Biegala, 2019). In neurons deprived of STIM2, the amount of mushroom dendritic spines, which are vital for memory storage, was decreased (Sun et al., 2014; Garcia-Alvarez et al., 2015; Yap et al., 2017). STIM2 also co-localizes with Ca^2+^/calmodulin-dependent protein kinase II (CaMKII) in dendritic spines and regulates its phosphorylation (Yap et al., 2017). STIM2-mediated SOCE sustained CaMKII activation and thus is important in the maintenance of dendritic spines. STIM2-Orai2-TRPC6 complexes regulate SOCE in mice hippocampal synapses and thus influence the number of dendritic spines. Orai-STIM2 complexes play an essential role in the formation of new synapses (Korkotian et al., 2017). All these studies demonstrate a vital role of STIM-mediated SOCE in both formation and maintenance of mushroom spines, and suggest its role in synaptic plasticity. Neuronal SOCE indeed takes part in long-term potentiation (LTP) and long-term depression (LTD), processes responsible for memory and learning (Serwach and Gruszczynska-Biegala, 2019). In Purkinje neurons (PNs), slow excitatory post-synaptic currents (EPSCs) were the result of TRPC3 activity. Lack of STIM1 resulted in no ER Ca^2+^ release and slow EPSCs (Hartmann et al., 2014). Thus, STIM1 is considered to refill the dendritic ER Ca^2+^ stores only under resting conditions. In resting cells, STIM1-mediated SOCE also caused an ubiquitination and degradation of Sp4 transcription factor (Lalonde et al., 2014). These results underlie an essential homeostatic function of STIM1-mediated SOCE in resting neurons.

### Autophagy

Autophagy is stimulated in response to various types of cellular stress, including ER stress, oxidative stress, starvation of nutrients and growth factors, hypoxia and mitochondrial damage (Kroemer et al., 2010). Moderate ER stress can improve the ability of the ER to process unfolded or misfolded proteins and maintain cell survival. However, if the stress is prolonged or extensive, homeostasis within the cell is disrupted leading to apoptosis, which is primarily mediated by Ca^2+^ overload, or autophagy (Berridge et al., 2000; Bernales et al., 2006; Ding et al., 2007; Høyer-Hansen and Jäättelä, 2007). Autophagy, e.g., caused by oxygen and glucose deprivation and reoxygenation, may maintain cellular homeostasis, but its excessive level may lead to autophagic neuronal necrosis and apoptosis (Ahsan et al., 2019; Zhou et al., 2019). Differences in cytosolic Ca^2+^ levels associated with autophagy and apoptosis have been demonstrated in several cell lines. Calcium is mostly considered as an activator of autophagy, but there are some reports that Ca^2+^ suppresses autophagy (Høyer-Hansen et al., 2007; Cárdenas et al., 2010; Law et al., 2010; Parys et al., 2012; Wong et al., 2013).

However, the role of STIM1/Orai1 in autophagy and apoptosis in the CNS is still unclear, and there is not much work on the subject, thus underscoring the need for further research in this field. Proteasome inhibitors MG-132 and LA promoted the autophagy-mediated degradation of STIM1 and STIM2 and thus reduced SOCE in neurons (Kuang et al., 2016). The opposite is true in HEK293 cells where the stability of STIM1 was not affected by proteasome inhibitors, although thapsigargin-induced surface levels of STIM1 and SOCE were increased in cells pretreated with MG-132 (Keil et al., 2010). These differences are likely to be due to the different conditions for treating cells with protease inhibitors and may also exist depending on the type of cells used. Further, Kondratskyi et al. demonstrated that, in prostate cancer cells, SOCE inhibitor (ML-9) stimulates autophagosome formation and inhibits autophagosome degradation independent of SOCE and STIM1 (Kondratskyi et al., 2014). On the other hand, in prostate cancer cells (DU145 and PC3), resveratrol has been proposed to induce autophagy by regulating the function of STIM1 and then SOCE. Indeed, STIM1 overexpression restores resveratrol-induced reduction of SOCE as well as autophagic cell death induced by ER stress (Selvaraj et al., 2016). In line with this, STIM1 and SOCE have been shown to positively regulate oxidized low-density lipoprotein-induced autophagy in endothelial progenitor cells (Yang et al., 2017). Recently, hypoxia-induced Ca^2+^ release from the ER in neuron-like PC12 cells was modulated by STIM1/Orai1 (Hu et al., 2020). In addition, STIM1/Orai1 signaling induced by α2-adrenergic receptor agonist, dexmedetomidine, following hypoxia was mediated by a decrease of [Ca^2+^]_i_, leading to a reduction of autophagy. The results suggest that dexmedetomidine may have neuroprotective effects against oxidative stress, autophagy, and neuronal apoptosis after oxygen-glucose deprivation and reoxygenation injury through modulation of Ca^2+^-STIM1/Orai1 signaling (Hu et al., 2020). In turn, STIM1 has been shown to be not essential in hypoxia-mediated autophagy in both SHSY-5Y and HSG cells (Sukumaran et al., 2015). The available data suggest that STIMs influence autophagy differently depending on cell type and triggers of autophagy.

### Neurodegenerative Diseases

**Figure 2:**
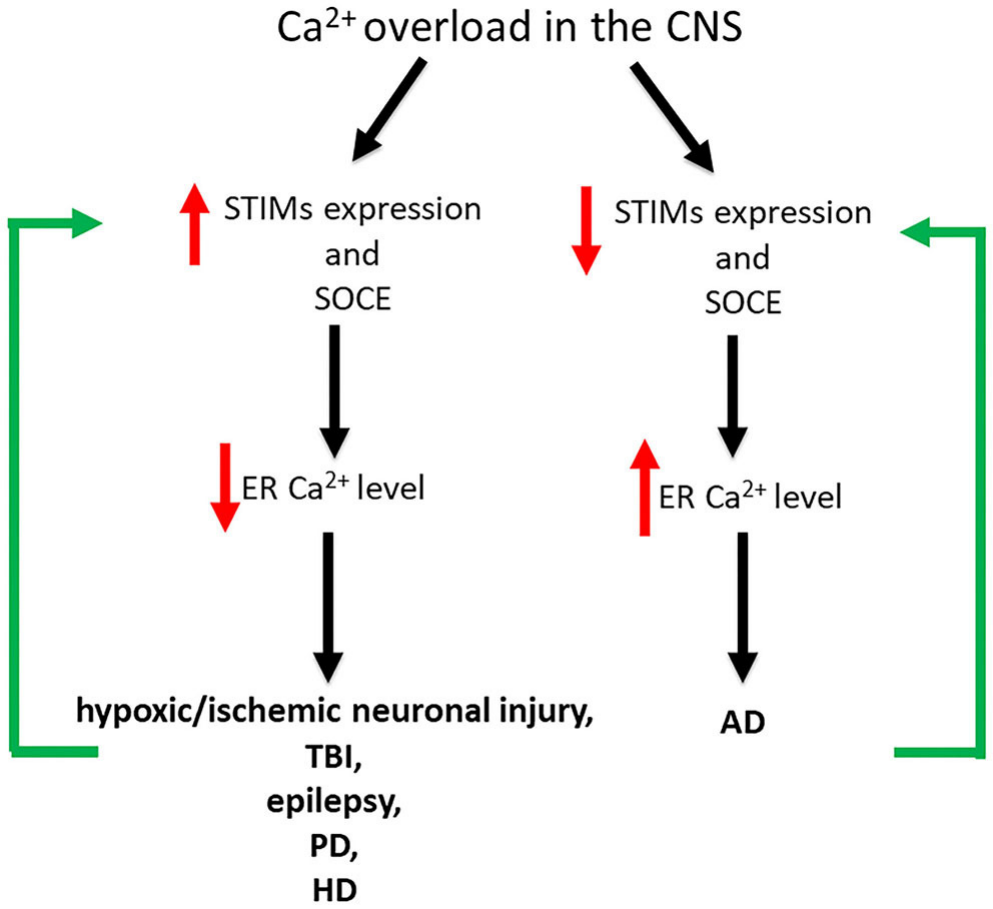
Impact of Ca^2+^ overload on STIM expression level and SOCE in the development of CNS disorders. In hypoxic/ischemic neuronal injury, TBI, epilepsy, PD, and HD Ca^2+^ overload is associated with increased STIM expression level and SOCE and thus decreased ER Ca^2+^ level. Contrary, in AD Ca^2+^ overload results in decreased STIM expression level and SOCE and thus increased ER Ca^2+^ level. SOCE blockage and STIM downregulation seem to be neuroprotective in hypoxic/ischemic neuronal brain injury, epilepsy, TBI, HD, and PD, while in AD increased expression level of STIMs and SOCE enhancement appear to be neuroprotective.

Dysregulation of neuronal SOCE and changes in STIM expression levels are associated with various pathological conditions of the CNS such as hypoxic/ischemic neuronal injury, TBI, epilepsy, AD, PD and HD (Figure 2; Serwach and Gruszczynska-Biegala, 2019). Many studies have demonstrated the role of STIM2 in hypoxic/ischemic neuronal injury (Soboloff et al., 2006b; Vig et al., 2006; Berna-Erro et al., 2009). Hippocampal neurons, both in slices and in culture, showed reduced ER Ca^2+^ level during hypoxia, and STIM2 reduced Ca^2+^ overload during ischemic challenge (Berna-Erro et al., 2009). *Stim2* knockout (KO) mice were better protected against cerebral ischemia (Berna-Erro et al., 2009). Thus, it seems that the absence of STIM2 may potentially constitute a protective strategy against stroke. STIM1 and STIM2 have also been implicated in epilepsy as they are up-regulated both in CA1 and CA3 regions of chronic epileptic mice (Steinbeck et al., 2011). Non-selective SOCE inhibitors rhythmized epileptic burst activity in epileptic hippocampal slices, suggesting that SOCE blockage may potentially bring positive effect in patients with epilepsy. STIM2 has also been shown to be overexpressed after TBI (Rao et al., 2015). The downregulation of STIM2 improved neuronal survival in models of TBI, decreasing neuronal apoptosis and preserving neurological function by alleviating mitochondrial disfunction and Ca^2+^ overload. STIM2 downregulation not only decreased Ca^2+^ release from the ER, but also reduced SOCE and dropped mitochondrial Ca^2+^ level, restoring its morphology and function. Downregulation of STIM2 has a neuroprotective effect and may be a target in TBI treatment (Rao et al., 2015). Dysregulation of SOCE also contributes to PD. A neurotoxin, which mimics PD *in vitro*, decreased level of TRPC1 and its interaction with STIM1, thus increasing neuronal death. Pharmacological inhibition of SOCE appears to be neuroprotective representing a potential target for PD drug discovery (Pchitskaya et al., 2018). In HD transgenic mice, over-activation of synaptic SOCE and enhancement of STIM2 expression resulted in the disruption of dendritic spines. STIM2 knockdown has been shown to normalize SOCE and prevent loss of dendritic spines. It seems that pharmacological modulation of SOCE and its components have neuroprotective effects in HD patients. On the other hand, in mice models of AD, impairment of SOCE and reduction of synaptic STIM2 proteins contributed to the destabilization of dendritic spines (Sun et al., 2014; Zhang et al., 2016). Since stabilization of dendritic spines is considered to prevent memory loss in AD patients, the modification of STIM proteins and SOCE may be a potential therapeutic target in the treatment of memory loss in these patients.

Changes in neuronal SOCE may vary among pathological states of the CNS. SOCE blockage and STIM downregulation seem to be neuroprotective in hypoxic/ischemic neuronal brain injury, epilepsy, TBI, HD and PD, while in AD STIMs and SOCE appear to be neuroprotective (Figure 2).

Misfolded proteins and the associated ER stress are common features of some neurodegenerative diseases such as PD, AD and HD. These properties can further induce autophagy or apoptosis in neurons (Ghavami et al., 2014; Remondelli and Renna, 2017). Given the high sensitivity of neurons to ER Ca^2+^ store disturbances, STIM and SOCE have been proposed as potential targets for neuroprotection by reversing ER and mitochondrial stress-induced damage. Interestingly, blockade of SOCE reduced apoptosis mediated by oxidative stress in hippocampal neuronal HT-22 cells (Rao et al., 2013). Hawkins et al. demonstrated that in lymphocyte cells oxidative stress favors STIM1 trafficking and puncta formation, which confirms that STIM1 is regulated by the redox state (Hawkins et al., 2010). In turn, the formation of the STIM1 puncta, their translocation to the PM and the subsequent SOCE in HEK cells were disrupted by mitochondrial depolarization in mitofusin 2 dependent manner. These effects have been shown to be overcome by overexpression of STIM1 (Singaravelu et al., 2011). Consequently, STIM1 in 401L neuroblastoma cells provided protection against ER stress and mitochondrial oxidative stress causing cell death (Zhang and Thomas, 2016). Experiments performed on embryonic fibroblasts also reported that STIM1 rescue protected from oxidative stress and enabled cell survival by impairing the translocation of the apoptosis-inducing factor into the nucleus (Henke et al., 2012). All these findings suggest that STIM may indeed provide protection against cell death mediated by the ER and oxidative stress, which often precede neurodegeneration.

## STIM-Binding Ca^2+^ Channels

**Figure 3:**
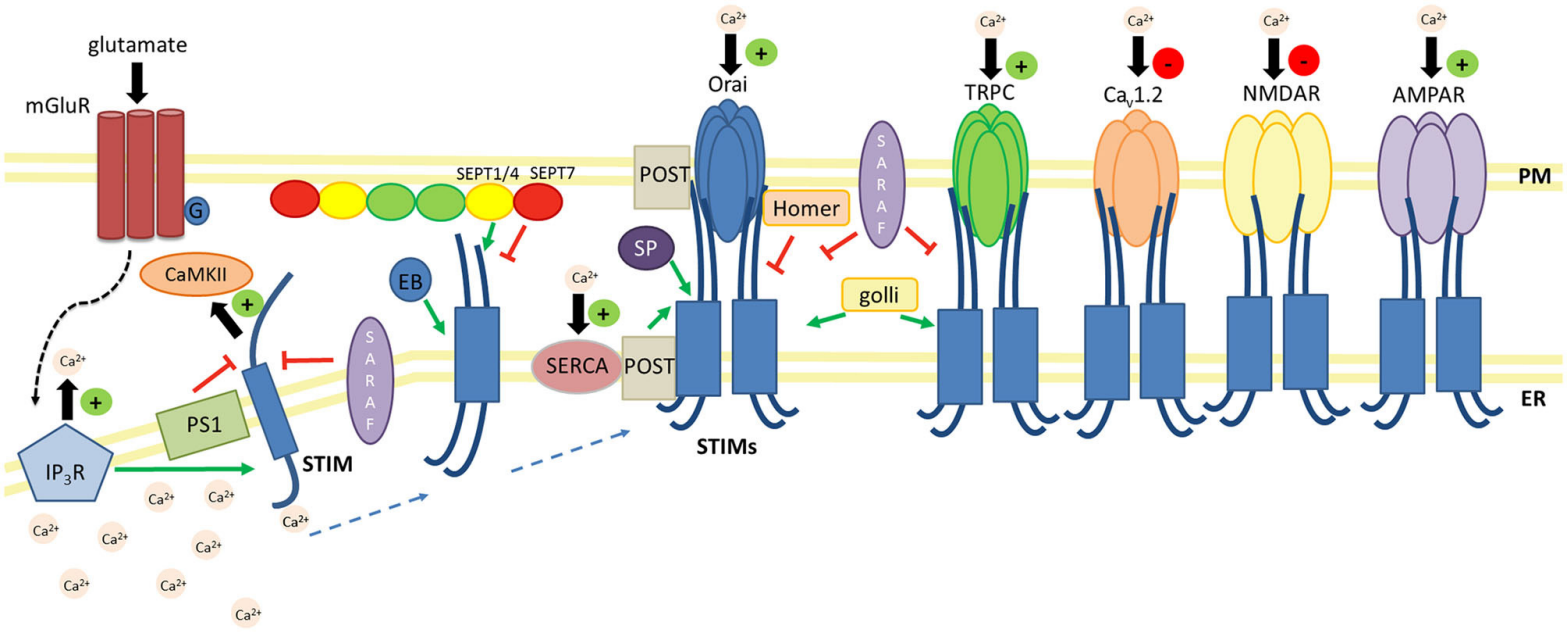
Schematic overview of key regulators and effectors of STIM proteins in the CNS. Negative regulators (—|): SARAF prevents STIM1 activation and inhibits the STIM1-Orai association. SARAF silencing increases TRPC1-mediated Ca^2+^ entry. The PS1–γ-secretase complex cleaves the STIM1 transmembrane domain. Homer1a dissociates the STIM1-Orai1 complex. Lower dSEPT7 expression increases the amount of dSTIM-dOrai clusters. Positive regulators (→): The glutamate-mediated activation of mGluRs results in Ca^2+^ release from ER stores via IP_3_Rs and activates STIM-Orai coupling. EB3 forms complexes with STIM2, which promotes the formation of mushroom spines in hippocampal neurons. SEPT1/4 regulates the number of ER-PM junctions and enhances STIM1-Orai1 interactions. The STIM1-POST complex binds to SERCA and promotes ER Ca^2+^ refilling. Golli proteins interact with STIM1 and TRPC1 and thus enhance SOCE. SP interacts with STIM and Orai and determines synaptic plasticity. Positive effectors (+): STIM proteins increase Ca^2+^ influx via Orai, TRPC, and AMPARs. STIM2-mediated SOCE activates CaMKII and thus stabilizes mushroom spines. Negative effectors (–): STIM proteins decrease Ca^2+^ influx via L-type VGCCs and NMDARs.

According to the current state of knowledge, neuronal STIM proteins regulate both CRAC and SOC. Other channel proteins, such as L-type voltage-gated Ca^2+^ channels (VGCCs; Ca_v_1.2, Ca_v_1.3) and receptor/ligand-activated Ca^2+^ channels (AMPARs and NMDARs), couple or engage in an interplay with STIMs in the CNS in a modulatory way as part of SOCE signaling (Figure 3).

### Orai

As mentioned above, emptying ER Ca^2+^ stores causes STIM protein oligomerization and translocation of the oligomers toward the PM where they form complexes, known as puncta, with an Ca^2+^ selective ion channel protein, Orai1. We previously showed that the depletion of Ca^2+^ from the ER by thapsigargin, a selective SERCA inhibitor, increased the number of puncta-like structures with Yellow Fluorescence Protein (YFP)-STIM1 and Orai1 but not those with YFP-STIM2 and Orai1 (Klejman et al., 2009; Gruszczynska-Biegala et al., 2011). In contrast, a reduction of extracellular Ca^2+^ levels with ethylene glycol-bis(β-aminoethyl ether)-*N*,*N*,*N*′,*N*′-tetraacetic acid (EGTA) triggered the puncta formation of both YFP-STIM1/Orai1 and YFP-STIM2/Orai1. Other results showed that endogenous STIM1 and STIM2 can interact with Orai1, which was observed in a co-immunoprecipitation assay and *in situ* proximity ligation assay (PLA; Gruszczynska-Biegala and Kuznicki, 2013). The higher association between endogenous STIM2 and Orai1 in cortical neurons occurred in the presence of BAPTA-AM, membrane permeable Ca^2+^ chelator, and in a low-Ca^2+^ medium but not in the presence of thapsigargin. When SOCE was induced, the greatest number of PLA signals that corresponded to integrated STIM1 and Orai1 puncta was visible. The interaction between them was quantified and correlated well with the number of exogenous complexes that formed under the same conditions (Klejman et al., 2009; Gruszczynska-Biegala et al., 2011; Gruszczynska-Biegala and Kuznicki, 2013).

We can conclude that STIM1 and STIM2 can activate Orai1 channels and play different roles in neuronal SOCE (Berna-Erro et al., 2009; Klejman et al., 2009; Keil et al., 2010; Gruszczynska-Biegala et al., 2011; Gruszczynska-Biegala and Kuznicki, 2013; Sun et al., 2014; Majewski and Kuznicki, 2015). In rat cortical neurons, STIM1 mainly forms complexes with Orai1 and activates SOCE only after Ca^2+^ is completely emptied from the ER. This demonstrates its role in maintaining the level of Ca^2+^ in the ER. In contrast, STIM2 forms a hetero-complex with Orai1 to allow the regulation of resting intracellular Ca^2+^ levels and activation of constitutive Ca^2+^ influx after a slight decrease in Ca^2+^ levels in the ER.

In the rat cortex, SOCE is mainly mediated by Orai1-STIM1 complexes. In the mouse brain, in contrast, it is triggered either by Orai2 and STIM2 (cortex and hippocampus; Berna-Erro et al., 2009; Sun et al., 2014) or by Orai2 and STIM1 (cerebellum; Hartmann et al., 2014). Likewise, STIM2-gated SOC channels in dendritic mushroom spines are formed by a complex of Orai2 and TRPC6 (Zhang et al., 2016; Popugaeva et al., 2020). In turn, both STIM1 and STIM2 are involved in SOCE in sensory neurons in dorsal root ganglia. Moreover, both Orai1 and Orai3 contribute to SOCE in these neurons, where they may form homomultimers to mediate SOCE (Wei et al., 2017).

In addition to a canonical function as an ER Ca^2+^ level refilling toolkit, STIM1–Orai1-mediated SOCE was also shown to regulate gene expression and proliferation in mouse and human neural progenitor cells (NPCs) and thus is thought to be a key regulator of neurogenesis in mammalian cells (Somasundaram et al., 2014; Gopurappilly et al., 2018). The knockdown of Orai1 or STIM1 diminishes SOCE in NPCs. SOCE is not observed in NPCs from transgenic mice that lack Orai1 or STIM1 or in knock-in (KI) mice that express a Orai1 mutant. The deletion or suppression of STIM1 and Orai1 diminishes the proliferation of embryonic and adult NPCs both *in vitro* and *in vivo* in the subventricular zone (SVZ) in the adult mouse brain (Somasundaram et al., 2014). Domenichini et al. showed that SOCE in SVZ cells is mediated not only by STIM1 and Orai1 but also by TRPC1 (Domenichini et al., 2018). The pharmacological blockade of this process in mouse SVZ cells decreases proliferation and impairs self-renewal by shifting the type of cell division from symmetric to asymmetric, thereby reducing stem cell population (Domenichini et al., 2018). In human NPCs, SOCE has been shown to be significantly attenuated by the short-hairpin RNA/micro RNA targeting of STIM1 (Gopurappilly et al., 2018). Gopurapilly et al. investigated global gene expression in human NPCs with *STIM1* knockdown and showed that signaling pathways that are associated with DNA replication and cell proliferation were downregulated, whereas post-synaptic cell signaling was upregulated in these cells (Gopurappilly et al., 2018). To understand the functional relevance of these gene expression alterations, these authors also measured the self-renewal capacity of NPCs with STIM1 knockdown and found a substantially smaller neurosphere size and number and a decrease in differentiation toward cells with a neuronal lineage. These findings demonstrate that STIM1-mediated SOCE in human NPCs regulates gene expression alterations, which is likely to modulate the differentiation and self-renewal of NPCs (Gopurappilly et al., 2018).

STIM1 and ORAI1 have been shown to be involved in Ca^2+^ signaling in both astroglia and glioblastoma cells. The knockdown of both STIM1 and Orai1 or Orai1 alone resulted in a reduction of SOCE in rat astrocytes (Moreno et al., 2012). The silencing of STIM1 or Orai1 was shown to reduce SOCE and CRAC currents in human glioblastoma cells (Motiani et al., 2013). Surprisingly, Ronco et al. found that Orai3 but not Orai1 is a dominant Orai homolog in astroglia (Ronco et al., 2014). Recent studies ascribed a role to STIM1, Orai1, and Orai3 in astroglial SOCE (Gao et al., 2016; Kwon et al., 2017). Additionally, the activation of SOCE in spinal astroglia promotes the production of proinflammatory cytokines, such as tumor necrosis factor α (TNF-α) and interleukin-6 (IL-6). The production of TNF-α and IL-6 was decreased by the knockdown of Orai1 or STIM1 (Gao et al., 2016). Interestingly, Orai1 and STIM2 knockdown minimized lipopolysaccharide (LPS)-induced TNF-α and IL-6 production (Gao et al., 2016). This research may provide a basis for assessing SOCE and its components for the treatment of chronic pain and other neurological diseases that are associated with astroglial overactivation.

Studies that focused on the optic nerve in mice identified Orai1 and STIM1 but not STIM2 in astrocytes, whereas Orai1, STIM1, and STIM2 in oligodendrocytes suggested that STIM1 may be localized in the cell soma, and STIM2 may be localized in myelin (Papanikolaou et al., 2017).

In cultured rat microglia, SOCE is mediated by Orai channels rather than TRPC channels (Ohana et al., 2009). Siddiqui et al. identified high levels of Orai1 and STIM1 in microglial podosomes, structures that are responsible for cell motility (Siddiqui et al., 2012). Another research group confirmed the expression of STIM1, Orai1, Orai2, and Orai3 in cultured mouse microglia and showed that the downregulation of STIM1 and Orai1 reduced SOCE in these cells (Heo et al., 2015). Michaelis et al. studied the role of Orai1 and STIM proteins in microglia using cells that were obtained from knockout mice (Michaelis et al., 2015). The results showed that SOCE was reduced in the absence of Orai1 or STIM proteins. SOCE was nearly absent in *Stim1*^−/−^ microglia and substantially reduced in *Orai1*^−/−^ microglia, whereas a less pronounced effect was observed in *Stim2*^−/−^ microglia (Michaelis et al., 2015). Orai1 and STIM1 appear to be major components of microglial SOCE, and STIM2 is also a constituent of this signaling pathway in these cells (Kraft, 2015). Interestingly, recent studies showed that STIM1- and Orai1-mediated SOCE regulate phagocytic activity and cytokine release in primary murine microglia (Heo et al., 2015). Phagocytic activity, as well as LPS stimulation-mediated proinflammatory cytokine release (e.g., TNF-α and IL-6), was inhibited by SOCE inhibitors and STIM1 and Orai1 knockdown (Heo et al., 2015). This research suggests that STIM1 may be a new regulatory target for the prevention of an excessive proinflammatory response of microglia in neurodegenerative disorders.

### TRPC Channels

In addition to Orai activation, STIM proteins may cause Ca^2+^ influx via TRPC channels, which are found in cells from all regions of the brain and spinal cord, with high TRPV (TRP channel subfamily V), TRPC, and TRPM (TRP channel subfamily M) expression (Verkhratsky et al., 2014). Nevertheless, TRPC1 and Orai1 activation is mediated by different STIM1 domains. TRPC1 is involved in SOCE, but like the other TRPC channels, it is unable to generate a current that resembles Ca^2+^-selective I_CRAC_ (Albarrán et al., 2016). TRPC1 function depends on Orai1-mediated Ca^2+^ influx, which triggers the recruitment of TRPC1 into the PM, where it is activated by STIM1. TRPC1 is thought to modify the initial Ca^2+^ signal that is caused by Orai1 activation (Ambudkar et al., 2017).

TRPC1-mediated SOCE is essential for neuronal survival (Wang and Poo, 2005; Bollimuntha et al., 2006; Selvaraj et al., 2012). The STIM1-TRPC1 interaction is thought to be neuroprotective in both *in vitro* and *in vivo* models of PD (Selvaraj et al., 2012; Sun et al., 2017, 2018). In the human SH-SY5Y neuroblastoma cell line, SOCE mainly depends on the activation of TRPC1. A neurotoxin that caused the selective loss of dopaminergic neurons (DNs) in the substantia nigra pars compacta (SNpc) decreases TRPC1 expression, the TRPC1-STIM1 interaction, and SOCE but not Orai expression (Selvaraj et al., 2012). TRPC1 overexpression prevents the neurotoxin-mediated loss of SOCE and decreases ER Ca^2+^ levels and the unfolded protein response (UPR). Additionally, TRPC1-mediated Ca^2+^ entry activates the neuroprotective AKT pathway. STIM1 or TRPC1 but not TRPC3 silencing increases the UPR. Consistent with these results, *Trpc1*^−/−^ mice have a higher UPR and lower number of DNs, similar to PD patients. The overexpression of TRPC1 in mice increased DN survival after neurotoxin treatment.

STIM1 was also shown to inactivate Ca^2+^ entry via VGCCs, which is detrimental to DNs. Thus, the STIM1-TRPC1 interaction was thought to inhibit Ca^2+^ influx via VGCC channels, thereby protecting DNs (Selvaraj et al., 2012). Subsequent research showed that TRPC1 regulates L-type VGCCs in SNpc neurons (Sun et al., 2017). The STIM1-TRPC1 interaction after store depletion reduced DN activity in wildtype (WT) but not *Trpc1*^−/−^ mice. In *Trpc1*^−/−^ SNpc neurons, L-type VGCC Ca^2+^ currents increased, STIM1-Ca_v_1.3 interactions were attenuated, and the number of DNs decreased. After TRPC1 activation, L-type Ca^2+^ currents and Ca_v_1.3 opening probability decreased, whereas they increased after STIM1/TRPC1 silencing. Additionally, store depletion increased the Ca_v_1.3-TRPC1-STIM1 association. TRPC1 appears to suppress Ca_v_1.3 activation proving that STIM1 is essential for DN survival (Sun et al., 2017).

Sun et al. showed that mesenchymal stem cell (MSC)-derived DNs, similar to native neurons, utilize TRPC1-mediated SOCE (Sun et al., 2018). Similar to SH-SY5Y cells, neurotoxin treatment in MSC-derived DNs decreased TRPC1 expression and SOCE. TRPC inhibition alleviated dopamine release and MSC-derived DN viability. These results indicate that ER Ca^2+^ levels that are maintained by TRPC1-mediated SOCE are neuroprotective. Neurotoxin exposure may cause alterations of SOCE and TRPC1-mediated Ca^2+^ homeostasis that may further induce ER stress and the UPR, leading to neurodegeneration. These results demonstrate that MSC-derived DNs are similar to native DNs, which potentially broadens the prospect of their usage for regenerative therapy in PD patients (Sun et al., 2018).

STIM1 and TRPC have also been shown to be involved in SOCE in astroglia. Antisense oligonucleotides that targeted the *Trpc1* gene reduced SOCE in murine astrocytes (Golovina, 2005). An anti-TRPC1 antibody lessened SOCE in rat astrocytes (Malarkey et al., 2008). In spinal astrocytes, SOCE was predominantly subserved by TRPC3 (Miyano et al., 2010). Some studies ascribed these differences between Orai- and TRPC-mediated SOCE to the stage of astroglial development, suggesting that SOCE in immature and maturating astroglia is predominantly mediated by Orai, whereas SOCE in mature cells is predominantly mediated by TRPC1 (Kettenmann and Bruce, 2013; Verkhratsky and Parpura, 2014). Another study speculated that Orai1 and Orai3 are expressed in astroglial cells with abundant SOCE, whereas TRPC1 is restricted to astroglia where this process is attenuated (Kwon et al., 2017). STIM proteins, Orai1, and TRPM3 were identified as constituents of SOCE in astrocytes and oligodendrocytes of the mouse optic nerve (Papanikolaou et al., 2017). The developmental downregulation of Orai1 is consistent with TRPC channels as major components of mature astrocytes and oligodendrocytes, suggesting a potential role for Orai/STIM SOCE in immature glia and TRPM3 in mature glia (Verkhratsky et al., 2014).

Müller glia, a type of retinal glial cell, expresses STIM1 and requires the synergistic activation of both TRPC1 and Orai channels. The precise mechanism by which Orai and TRPC1 are activated by STIM1 has not been ascertained in these cells (Molnár et al., 2016).

### VGCCs

VGCCs are Ca^2+^ channels that are present primarily in electrically excitable cells (as neurons), known as transducers of electrical activity that enable Ca^2+^ influx in response to subthreshold depolarizing stimuli or action potentials (Harraz and Altier, 2014). They are assembled from the pore-forming α1 subunit and accessory β and α2δ-like subunits (Heine et al., 2020). Various isoforms of the α1 subunit differ in voltage and Ca^2+^ sensitivity, which defines the specific kinetic properties of the channel. Thus, VGCCs are classified into low and high voltage-activated. The accessory β and α2δ-like subunits play a role in membrane trafficking and the modulation of kinetic properties of high voltage-activated Ca^2+^ channels (Campiglio and Flucher, 2015; Brockhaus et al., 2018). α2δ-like subunit and chemotaxis receptor domain containing 1 (Cachd1) alter the kinetic properties and surface expression of VGCCs (Cottrell et al., 2018; Dahimene et al., 2018). Cachd1 also impacts the gating and trafficking of low voltage-activated Ca^2+^ channels (Cottrell et al., 2018).

In excitable cells, VGCCs are the main route of Ca^2+^ entry in response to depolarizing stimuli. The main VGCC subtype that is present in neuronal, cardiac, and smooth muscle cells is Ca_v_1.2, whereas Ca_v_1.3 is the predominant subtype in DNs (Sun et al., 2017). Both VGCC subtypes were shown to be suppressed by STIM1 (Park et al., 2010; Wang et al., 2010; Harraz and Altier, 2014; Sun et al., 2017, 2018). Two independent studies of excitable cells showed that ER Ca^2+^ store depletion alleviates depolarization-mediated Ca_v_1.2 activity, whereas the Ca_v_1.2 response increases after functional impairments in STIM1 (Park et al., 2010; Wang et al., 2010). STIM1-Ca_v_1.2 interactions are directly mediated by the SOAR domain (i.e., the same domain that activates SOCs) of STIM1 and C-terminus of the Ca_v_1.2 α1 subunit (Harraz and Altier, 2014; Pascual-Caro et al., 2018). The influence of STIM1 on VGCCs is also associated with an increase in channel internalization from the PM. Despite reporting similar results, two studies suggested different inhibitory mechanisms. Park et al. proposed a mechanism that involves the attenuation of VGCC expression, whereas Wang et al. suggested a potential role for Orai1 in the inhibitory STIM1-Ca_v_1.2 interaction because the simultaneous inhibition of both Orai1 and STIM1 was necessary to suppress Ca_v_1.2 activity (Park et al., 2010; Wang et al., 2010).

The ability of STIM1 to regulate Orai1 and Ca_v_1.2 is tissue specific. STIM1 appears to stimulate SOCE in non-excitable cells and inhibit VGCCs in excitable cells (Harraz and Altier, 2014). STIM1 was also shown to control the plasticity of L-type VGCC-dependent dendritic spines (Dittmer et al., 2017; Sather and Dittmer, 2019). The activation of neuronal STIM1 induces changes in the ER structure, which depends on L-type VGCCs. The NMDAR activation of L-type VGCCs triggers Ca^2+^ release from the ER, which in turn causes STIM1 aggregation and its coupling with L-type VGCCs and then inhibits the activation of this channel, thus increasing ER spine content and stabilizing mushroom spines (Dittmer et al., 2017). STIM1 deficiency is associated with AD and triggers SH-SY5Y cell death by upregulating Ca_v_1.2 (Pascual-Caro et al., 2018). Thus, STIM1 KO cells may constitute an *in vitro* model to study the pathogenesis of AD and may be useful for understanding the role of STIM1 in neurodegeneration. In turn, as mentioned in section TRPC Channels above, TRPC1 in DNs facilitates STIM1-Ca_v_1.3 interactions to suppress Ca_v_1.3 activity, thereby reducing apoptosis and protecting DNs against neurotoxin-induced insults that lead to PD (Sun et al., 2017).

### AMPA Receptors

AMPARs belong to the family of ionotropic glutamate receptors. They are thought to be the most significant mediators of excitatory neurotransmission in the CNS (Rogawski, 2011). They are assembled from four subunits (GluA1-4) and are mainly permeable to Na^+^ and K^+^ and to Ca^2+^ to a lesser extent. The subunit composition of AMPARs varies depending on the stage of development, region, and cell type (Song and Huganir, 2002). Phosphorylation of the GluA1 C-terminal tail regulates activity-dependent synaptic transport and channel features of the receptor (Esteban et al., 2003). Ser-831 and Ser-845 are two phosphorylation sites of GluA1 that have been well-characterized. Phosphorylation at Ser-831 by CaMKII and protein kinase C (PKC) regulates channel conductance, whereas cyclic adenosine monophosphate (cAMP)/protein kinase A (PKA)-dependent phosphorylation at Ser-845 enhances channel open probability and promotes AMPAR internalization (Derkach et al., 1999). The PKA-dependent phosphorylation of GluA1 depends on the PKA scaffold AKAP150, which places PKA in proximity to its synaptic targets (Garcia-Alvarez et al., 2015) and cAMP-mediated dissociation of the regulatory subunit (rPKA) from the catalytic subunit (cPKA; Garcia-Alvarez et al., 2015). Both Ser-845 and Ser-831 phosphorylation sites play a role in LTP and LTD, forms of synaptic plasticity that are responsible for learning and memory (Esteban et al., 2003; Makino and Malinow, 2011).

Interestingly, STIM2 was shown to interact with AMPARs through a mechanism that is not associated with SOCE (Garcia-Alvarez et al., 2015). Recent research showed that STIM2 induces the cAMP/PKA-dependent delivery of GluA1 to the PM (Garcia-Alvarez et al., 2015). These authors suggested that STIM2 couples PKA to AMPARs and promotes the phosphorylation of GluA1 at Ser-845. They revealed a strong interaction between STIM2 and cPKA and weak STIM2 binding to rPKA and AKAP150, which may clarify the mechanism of interaction. Surprisingly, STIM2 and the phosphorylation of GluA1 at Ser-831 are negatively correlated. In STIM2-silenced neurons, phosphorylation at Ser-831 is enhanced. These findings indicate that STIM2 regulates the phosphorylation of GluA1 at both Ser-845 and Ser-831 but in a different manner. In turn, STIM1 overexpression was shown to increase GluA1 phosphorylation at Ser-845 in hippocampal synaptoneurosomes (Majewski et al., 2017).

Our previous study found that STIM proteins in primary rat cortical neurons may also interact with AMPARs in a SOCE-dependent manner, meaning that when ER Ca^2+^ stores are depleted, Ca^2+^ may enter through Orais, TRPC channels, and AMPARs (Gruszczynska-Biegala et al., 2016). The SOCE inhibitors ML-9 and SKF96365 decreased AMPA-induced Ca^2+^ influx, and the competitive AMPAR antagonists CNQX and NBQX inhibited SOCE. The induction of SOCE by thapsigargin resulted in AMPAR activation either directly through the recruitment of AMPARs to the PM or indirectly through unknown mechanisms. We also confirmed that both STIM1 and STIM2 proteins directly interacted with the GluA1 and GluA2 subunits of AMPARs. Moreover, STIM-AMPAR complexes appear to be located in ER-PM junctions (Gruszczynska-Biegala et al., 2016).

### NMDA Receptors

NMDARs are ligand-gated ion channels that mediate Ca^2+^ influx when activated by glutamate, the main excitatory neurotransmitter in the mammalian CNS. NMDARs are tetramers that are composed of two glycine-binding NR1 (GluN1) and two glutamate-binding NR2 (GluN2) subunits (Cull-Candy and Leszkiewicz, 2004). Synaptic NMDARs consist mainly of NR1-NR2A or NR1-NR2A-NR2B receptors, and somatic NMDARs consist mainly of NR1-NR2B (Cull-Candy and Leszkiewicz, 2004). Ca^2+^ influx through NMDARs plays an important role in neuronal development, the formation of basal excitatory synaptic transmission, cell survival, and different forms of synaptic plasticity, such as LTP and LTD (Malenka and Bear, 2004; Nakazawa et al., 2004; Kerchner and Nicoll, 2008).

Pathologically high levels of glutamate and NMDA cause excitotoxicity by allowing high levels of Ca^2+^ to enter the cell (Sattler and Tymianski, 2000). Excessive NMDAR stimulation and prolonged increases in intracellular Ca^2+^ concentrations cause Ca^2+^ overload, which is considered a main cause of neuronal death in various neurodegenerative diseases that are associated with excitotoxicity, such as HD and AD (Marambaud et al., 2009; Pchitskaya et al., 2018; Serwach and Gruszczynska-Biegala, 2019). Attenuating intracellular Ca^2+^ overload is thus essential for limiting neuronal cell death under neuropathological conditions.

In hippocampal pyramidal neurons, SOCE can be activated by synaptic NMDAR stimulation, thus demonstrating its involvement in synaptic plasticity, such as LTP (Baba et al., 2003; Dittmer et al., 2017). Interestingly, glutamate release from neuronal terminals and NMDAR activation also induce SOCE in the PM of adjacent astrocytes possibly involving astrocytic mGluR5 (Lim et al., 2018). We recently reported that NMDARs contribute to Ca^2+^ influx in SOCE in rat cortical neurons (Gruszczynska-Biegala et al., 2020). The glutamate depolarization of neurons activates Ca^2+^ influx through NMDARs and L-type VGCCs, releases Ca^2+^ from the ER (Simpson et al., 1995; Emptage et al., 1999, 2001; Dittmer et al., 2017), and aggregates and activates STIM1, which then triggers SOCE (Rae et al., 2000; Emptage et al., 2001; Dittmer et al., 2017). In recent work, we found that endogenous STIM1 and STIM2 interact *in situ* and *in vitro* and co-localize with endogenous NMDAR subunits in rat cortical neurons. Emptying Ca^2+^ from ER stores induces a decrease in the physical association between endogenous STIM1 and NR2B and between STIM2 and NR2A (Gruszczynska-Biegala et al., 2020). Additionally, STIMs were shown to modulate NMDA-evoked intracellular Ca^2+^ levels by interacting with them. These data suggest that STIM1 and STIM2 are negative regulators of NMDA-induced intracellular Ca^2+^ elevations in cortical neurons (Gruszczynska-Biegala et al., 2020), in which they have been shown to inhibit the activity of L-type VGCCs (Park et al., 2010; Wang et al., 2010; Dittmer et al., 2017).

## STIM Interacting Proteins

Most of the aforementioned studies on STIM function in the CNS focused on its canonical function in ER Ca^2+^ store refilling that results from the activation of SOCE. Recently, an increasing number of proteins have been reported to play a vital role in the regulation of Orai1-, TRPC-, and STIM1-associated Ca^2+^ signaling in both a SOCE-dependent and SOCE-independent manner in the CNS. This section focuses on several of the most important partners of STIM proteins that may also contribute to essential molecular processes in the CNS (Figure 3).

### mGluRs

Group I metabotropic glutamate receptors (mGluRs) are widely distributed in the CNS and play a key role in synaptic transmission and plasticity. Excessive mGluR activation was shown in acute and chronic neurodegenerative disorders such as PD, AD, and HD (Crupi et al., 2019). The stimulation of group I mGluRs activates two signaling pathways (Hartmann et al., 2014). The first signaling pathway activates phospholipase C (PLC), thus inducing the formation of IP_3_, which interacts with IP_3_Rs to release Ca^2+^ from ER stores (Hartmann et al., 2014). The second signaling pathway involves the formation of slow excitatory post-synaptic potentials (EPSPs; Batchelor et al., 1994) and is mediated by TRPC3 (Hartmann et al., 2008).

STIM proteins also interact with TRPC3, and the STIM-mGluR association has been widely investigated. Hartman et al. showed that following mGluR1 activation, STIM1 proteins oligomerize and evoke SOCE through TRPC3 channels (Hartmann et al., 2008, 2014). Consistent with this, Ng et al. found that the activation of group I mGluRs with 3,5-dihydroxyphenylglycine (DHPG) in hippocampal neurons stimulated STIM1 oligomerization and its transport to the PM (Ng et al., 2011). Another research group showed that STIM1 in cerebellar PNs participates in mGluR1-dependent synaptic transmission and thus regulates cerebellar motor behavior (Hartmann et al., 2014). In mice with the PN-specific deletion of STIM1, mGluR1-dependent signaling was eliminated, and both IP_3_-dependent Ca^2+^ release from the ER and TRPC3-mediated EPSCs were attenuated. The disruption of these two pathways abolished cerebellar motor behavior (Hartmann et al., 2014). The role of STIM1 in synaptic plasticity was also investigated in hippocampal slices from adult transgenic Tg(STIM1)Ibd mice. STIM1 overexpression appears to disrupt mGluR LTD that is induced by DHPG (Majewski et al., 2017).

A recent analysis by Tellios et al. showed that in the absence of neuronal nitric oxide synthase (nNOS)-derived NO signaling along with the higher expression of mGluR1, STIM1 expression and cluster density are elevated in PNs (Tellios et al., 2020). These authors suggested that the overactivation of mGluR1 results in ER Ca^2+^ depletion and chronic STIM1 oligomerization. Because of the unlimited opening of TRPC3 channels, Ca^2+^ entry through STIM1-gated TRPC3 channels may be elevated, further leading to a reduction of dendritic spine integrity in PNs (Tellios et al., 2020). In contrast, under physiological conditions, nNOS/NO signaling maintains Ca^2+^ homeostasis in neurons by reducing its influx through mGluR1 and inducing the *S*-nitrosylation of STIM1. As STIMs and Orais as well as NMDARs and mGluRs are expressed in the microvascular endothelial cells of the brain (LeMaistre et al., 2012; Negri et al., 2020), we can suspect that these endothelial receptors may also be regulated by STIMs. Especially since the Ca^2+^ response to glutamate by activating mGluR1 and mGluR5 is initiated by endogenous Ca^2+^ release from the ER through IP_3_R_3_ and sustained by SOCE, resulting in a rapid NO release (Negri et al., 2020).

### Homer Family Proteins

Homer family proteins are post-synaptic scaffolding proteins that regulate glutamatergic signaling and intracellular Ca^2+^ concentrations in neurons (Chen et al., 2012). Homer was reported in neurological disorders, including AD, TBI, and schizophrenia (Luo et al., 2012). These proteins are divided into two major groups: short-form Homer1a proteins and long-form Homer1b/c, Homer2, and Homer3 proteins. Both groups have N-terminal EVH1 domain that is involved in protein interactions, and long-form proteins also have a C-terminal coiled-coil domain that is involved in self-association (Chen et al., 2012). Homer1b/c has been shown to alter SOCE through an association with STIM1 and Orai1 in human platelets. This interaction between STIM1, Homer1b/c, and Orai1 is enhanced by thapsigargin (Jardin et al., 2012). Thapsigargin also induces Homer1, STIM1, and L-type VGCC associations in HEK-293 cells (Dionisio et al., 2015).

In the CNS, SOCE antagonists and STIM1-targeted small-interfering RNA (siRNA) increase the expression of *Homer1a* mRNA and the amount of Homer1a protein (Li et al., 2013). The knockdown of Homer1a expression partially reverses this effect. Moreover, SOCE inhibition appears to protect neurons against oxidative stress through the upregulation of Homer1a expression (Li et al., 2013). SOCE inhibitors also prevented mitochondrial dysfunction and activation of mitochondrial apoptotic factors after MPP^+^ injury. Since mitochondrial dysfunction is thought to play a crucial role in PD, it seems that SOCE may be a potential target in the treatment of PD patients (Li et al., 2013).

Homer1a affects STIM1-Orai1 associations, inhibits SOCE, and alleviates glutamate-induced cell death after oxidative stress injury (Rao et al., 2016). Both thapsigargin-induced and glutamate-mediated STIM1-Orai1 associations are attenuated by Homer1a overexpression. After thapsigargin-induced ER Ca^2+^ store depletion, the association between STIM1 and Homer1a decreases, whereas no change occurs in the Homer1a-Orai1 association. These interactions between STIM1, Homer1a, and Orai1 are similar to interactions between STIM1 and Homer1b/c or STIM1, Homer1b/c, and Orai1 (Jardin et al., 2012; Dionisio et al., 2015; Rao et al., 2016). Therefore, Homer1a might dissociate STIM1-Orai1 complexes and downregulate SOCE through negative competition with Homer1b/c (Rao et al., 2016). The regulation of Homer1a and SOCE inhibition may be a potential therapeutic target for the treatment of neurological disorders, the etiology of which is associated with oxidative stress.

### SARAF

SOCE-associated regulatory factor (SARAF) is a 339-amino-acid type I transmembrane protein that has exceptionally high transcript levels in neuronal tissues (Palty et al., 2012). It is assembled from a single N-terminal transmembrane domain and a C-terminal domain that is located in the ER or PM (Palty et al., 2012; Albarran et al., 2016). The activation of SARAF involves the intraluminal region, whereas the interaction with SARAF engages the cytosolic region (Jha et al., 2013). Gene product of TMEM66, SARAF was identified as a biomarker linked to AD (Twine et al., 2011).

SARAF modulates STIM1-regulated Ca^2+^ entry, which includes both SOCE and Ca^2+^ influx through ARC channels (Palty et al., 2012; Albarran et al., 2016). SARAF prevents the spontaneous activation of STIM1, regulates STIM1–Orai1-mediated SOCE, facilitates the slow Ca^2+^-dependent inactivation of SOCE, and promotes STIM1 deoligomerization after Ca^2+^ store refilling (Palty et al., 2012; Jha et al., 2013). SARAF was also reported to modulate cytosolic and ER Ca^2+^ levels (Palty et al., 2012). The molecular mechanism of action of SARAF was described by Jha et al., who showed that SARAF interacts with the C-terminal inhibitory domain (CTID) of STIM1 to mediate the slow Ca^2+^-dependent inactivation (SCDI) of Orai1-forming CRAC (Jha et al., 2013). Under resting conditions, when intracellular Ca^2+^ stores are filled with Ca^2+^, CTID facilitates the access of SARAF to the SOAR to keep STIM1 in an inactive state, resulting in the inhibition of STIM1-Orai communication. Upon Ca^2+^ store depletion, SARAF dissociates from STIM1 to allow the activation of STIM1-Orai1 complexes at ER-PM junctions, thereby leading to SOCE (Jha et al., 2013).

SARAF is constitutively expressed in the PM. It was also shown to modulate Ca^2+^ entry through ARC channels in the SH-SY5Y neuroblastoma cell line (Albarran et al., 2016). ARC channels are heteropentameric complexes that consist of three Orai1 and two Orai3 subunits and PM-resident STIM1. The overexpression of SARAF in neuroblastoma cells attenuated the arachidonic acid (AA)-induced Ca^2+^ response, and the transfection of SARAF siRNA enhanced AA-stimulated Ca^2+^ influx via ARC channels. The results suggest that SARAF is a negative regulator of AA-induced Ca^2+^ signaling (Albarran et al., 2016).

SARAF was also shown to interact with TRPC1 in a STIM1-independent manner in both STIM1-defficient NG115-401L and endogenous STIM1-expressing SH-SY5Y neuroblastoma cells (Albarrán et al., 2016). The silencing of SARAF expression in STIM1-deficient cells increased TRPC1-mediated Ca^2+^ entry. In cells that endogenously expressed STIM1, the interaction between SARAF and TRPC1 was not associated with STIM1. This regulation of Ca^2+^ entry is thought to protect the cell from Ca^2+^ overload and adjust the influx of Ca^2+^, which was previously reported for the regulation of SOCE and ARC channels (Albarran et al., 2016). The silencing of SARAF expression did not influence TRPC6-mediated Ca^2+^ entry, in contrast to TRPC1, meaning that SARAF is unlikely to regulate the TRPC6 function. SARAF appears to play a negative regulatory role in both STIM1–Orai1- and STIM1–TRPC1-mediated Ca^2+^ entry by destabilizing STIM1/Orai1 complexes (Palty et al., 2012). Notably, a recent study reported a reduction of the expression of STIM1 and SARAF in the ischemic cortex, indicating that SARAF may be a new neuroprotective target for the treatment of stroke (La Russa et al., 2020).

### Septins

Septins are a class of evolutionary conserved GTPases that are assembled into hexameric or octameric complexes organized into linear filaments or other higher-order structures. They function as diffusion barriers and intracellular scaffolds in cells during various cellular processes (Mostowy and Cossart, 2012). The altered septin function may contribute to synaptic dysfunction in neurodegenerative diseases (Marttinen et al., 2015).

Septins were shown to regulate SOCE in both non-excitable mammalian cells (Sharma et al., 2013) and *Drosophila* neurons (Deb and Hasan, 2016, 2019; Deb et al., 2016, 2020). In *Drosophila*, septins are classified into several groups: dSEPT1, dSEPT2, dSEPT4, dSEPT5 and dSEPT7. The dSEPT7, dSEPT1, and dSEPT4 groups form linear hexamers. dSEPT1 and dSEPT4 occupy the central position, and dSEPT7 is localized in terminal positions of the hexamer. These hexamers are then linked and form linear septin filaments (Mostowy and Cossart, 2012; Mavrakis et al., 2014). The molecular mechanism of SOCE regulation and contribution of different subgroups of septins to the regulation of SOCE is complex.

The simultaneous knockdown of dSEPT1 and dSEPT4 reduced SOCE in *Drosophila* flight circuit neurons (Deb et al., 2016). The knockdown of these subgroups results in the loss of septin filaments and loss of the diffusion barrier, which has a negative influence on Orai activation by STIM (Deb and Hasan, 2016). dSEPT1 and dSEPT4 appear to function as positive regulators of SOCE in *Drosophila* neurons (Deb et al., 2016; Deb and Hasan, 2019). On the other hand, reduction of dSEPT7 had no significant influence on SOCE in *Drosophila* neurons. Nevertheless, the reduction of dSEPT7 in primary neurons that had low levels of *Drosophila* STIM1 (dSTIM1) improved SOCE (Deb et al., 2016). Additionally, STIM1 is necessary for SOCE through Orai channels also in SEPT7 knockdown human neural progenitor cells (hNPCs; Deb et al., 2020). In resting neurons with low dSEPT7 expression, the intensity of dSTIM and resulting dSTIM-dOrai clusters that are observed near the ER–PM region increased (Deb et al., 2016, 2020). Similar STIM1 and Orai1 reorganization was shown at the cell surface in SEPT7 knockdown neurons that were differentiated from hNPCs (Deb et al., 2020). Deb et al. suggested that the partial reduction of dSEPT7 leaves hexameric complexes intact but results in the formation of smaller septin filaments because filament elongation requires dSEPT7 (Deb et al., 2016). Shorter SEPT7 filaments support dSTIM migration to the peripheral ER in resting neurons, promoting Orai channel opening independently from either ER-Ca^2+^ store depletion or Ca^2+^ release through IP_3_Rs. Similarly, SEPT4 regulates the number of the ER-PM junctions and enhances STIM1-Orai1 interactions within junctions in human cells (Katz et al., 2019). Store-independent dOrai Ca^2+^ influx results in higher cytosolic Ca^2+^ concentrations in resting neurons (Deb and Hasan, 2016). The loss of dSEPT7 influences the constitutive activation of Orai channels in resting neurons by uncoupling septin heteromers from ER-PM junctions, thus allowing the STIM interaction with Orai (Deb et al., 2016, 2020).

Septins (e.g., dSEPT1, dSEPT4, and SEPT7) appear to perform antagonistic rather than synergistic functions in Orai channel activation by STIM. This discrepancy may be caused by a different assembly of septins during complex formation and by differences in subsequent filament structure (Deb and Hasan, 2019). Altogether, these results could link alterations of septin expression to impairments in STIM1-dependent SOCE in human neurodegenerative diseases.

### Golli Proteins

Golli proteins are isoforms of myelin basic protein (MBP) that are abundantly expressed in immune cells and the CNS (Paez et al., 2011). It is upregulated in adult OPCs and microglia in multiple sclerosis lesions (Filipovic et al., 2002). Myelin abnormalities have been implicated in neurodegenerative and neuropsychiatric diseases and Golli-MBP expression was increased in the aging brains (Siu et al., 2015). Golli proteins were shown to be components of remyelination that is caused by treatment with taxol in demyelinating transgenic mice, thus demonstrating their role in early stages of OPC proliferation and migration (Moscarello et al., 2002). Although no evidence suggests the occurrence of SOCE in oligodendroglia cells, this process was detected in OPCs and brain slices and shown to be mediated by STIM1 and TRPC1. Additionally, SOCE in these cells is positively modified by golli proteins that interact with STIM1 and TRPC1 (Paez et al., 2011). Changes in golli protein expression alter VGCCs and SOCs to mediate the migration and proliferation in OPCs that influence their maturation and survival (Paez et al., 2009a,b; Paez et al., 2007). Another study showed that golli protein overexpression increases the mitogen-stimulated proliferation of OPCs through the activation of SOCE, which is essential for cell division (Paez et al., 2009a). In OPCs, the proliferation of golli protein-KO cells was less robust, and the duration of the cell cycle increased. Golli proteins were also reported to increase apoptotic cell death, which was associated with an increase in Ca^2+^ influx through VGCCs (Paez et al., 2009a). Notably, the C-terminus of STIM1 was also shown to bind to MBP in a brain extract in a pull-down assay, which is likely an epitope that is shared with golli protein (Walsh et al., 2010).

### POST and SERCA

Several molecular mechanisms are responsible for the increase in cytosolic Ca^2+^ concentrations after cell depolarization, including SERCA, PMCA, NCX, and mitochondrial Ca^2+^ uptake. Among these mechanisms, SERCA and PMCA are regulated by STIM1, combined with an adaptor protein called partner of STIM1 (POST; Krapivinsky et al., 2011). After ER Ca^2+^ store depletion, the STIM1-POST complex binds to SERCA and keeps it close to Ca^2+^ entry sites on the PM to promote the refilling of ER Ca^2+^ stores (Krapivinsky et al., 2011). The STIM1-POST complex also inhibits PMCA activity, which is associated with Ca^2+^ outflow from the cytoplasm to the extracellular space (Ritchie et al., 2012). STIM1 appears to play opposing roles at the same time. However, SERCA contributes more to cytosolic Ca^2+^ clearance than PMCA, especially in PNs (Fierro et al., 1998). Thus, after PM depolarization, STIM1 reduces cytoplasmic Ca^2+^ concentrations. Additionally, Ryu et al. showed that STIM1 contributes to SERCA-dependent cytosolic Ca^2+^ clearance in the soma of firing PNs (Ryu et al., 2017). These authors proposed that the STIM1-POST complex may pull SERCA into the vicinity of VGCCs. If so, then SERCA could buffer Ca^2+^ influx more rapidly after depolarization, which can optimize the Ca^2+^-clearing and -buffering function of SERCA and prevent excessive cytosolic Ca^2+^ concentrations during repetitive firing (Ryu et al., 2017).

STIM1 mediates the sequestration of cytosolic Ca^2+^ ions by SERCA. It may also regulate neuronal excitability. Nevertheless, still unknown is whether this occurs only with high-firing neurons, such as PNs, or also with slow-firing neurons, such as pyramidal and cortical neurons (Ryu et al., 2017).

In this context it is worth noting that the function of SERCA can be also regulated by ER lumen residents calreticulin and ERp57 oxidoreductase (Li and Camacho, 2004). Thus, we suspect that these proteins may interact with STIM in neurons, especially since they are expressed therein (Coe and Michalak, 2010). ERp57 and STIM1 formed complexes *in vivo* and *in vitro* to inhibit STIM oligomerization and SOCE in mouse embryonic fibroblasts (Prins et al., 2011). Interestingly, in megakaryocytes from healthy individuals, calreticulin regulated SOCE activation through interaction with ERp57 and STIM1. In megakaryocytes from patients with mutated calreticulin, destabilization of the complex between calreticulin, ERp57 and STIM1 was observed, leading to enhanced SOCE and thus to abnormal cell proliferation (Di Buduo et al., 2020). ERp57 is associated not only with myeloproliferative neoplasms, but also with many disease states of CNS, such as prion disorders and AD, where ERp57 and calreticulin have been shown to prevent amyloid aggregation (Coe and Michalak, 2010). Similarly, SERCA-mediated Ca^2+^ dysregulation is associated with neuropathological conditions, such as affective disorders and neurogenerative diseases (Britzolaki et al., 2020).

### IP_3_Rs

Receptors that activate PLC cause the formation of IP_3_, which triggers both Ca^2+^ release from ER stores through IP_3_Rs and Ca^2+^ influx from the extracellular milieu, which is mediated by SOCE. IP_3_Rs are thought to regulate SOCE by mediating ER Ca^2+^ release. Under physiological conditions, store depletion causes STIM and IP_3_R accumulation near the PM, an association between STIM and Orai, and the activation of SOCE. In *Drosophila* neurons with mutant IP_3_Rs, SOCE was attenuated (Chakraborty et al., 2016; Deb et al., 2016) and this attenuation was reversed by STIM and Orai overexpression. The authors speculated that after ER Ca^2+^ store depletion in *Drosophila* neurons, IP_3_R translocation to the ER-PM junction triggers the coupling of STIM to Orai, leading to the activation of SOCE (Chakraborty et al., 2016).

The same research group also reported the enhancement of spontaneous Ca^2+^ influx from the extracellular milieu and loss of SOCE in *Drosophila* pupal neurons with mutant IP_3_Rs (Chakraborty and Hasan, 2017). Both spontaneous Ca^2+^ influx and the attenuation of SOCE were reversed by dOrai and dSTIM overexpression. Additionally, the expression of VGCCs decreased, and the expression of *trp* mRNAs and TRPC protein increased in mutant neurons, suggesting that these channels might be associated with the increase in spontaneous Ca^2+^ influx. Spontaneous Ca^2+^ influx likely compensates for the loss of SOCE in *Drosophila* IP_3_R mutant neurons and maintains intercellular Ca^2+^ homeostasis (Chakraborty and Hasan, 2017). The overexpression of dSTIM in insulin-producing neurons and aminergic neurons also improves SOCE and restores flight in a flightless *Drosophila* IP_3_R mutant (Agrawal et al., 2010). These authors suggested that IP_3_R-mediated Ca^2+^ release couples to SOCE via dSTIM/dOrai in *Drosophila* flight circuit neurons, thereby allowing dSTIM to compensate for impairments in IP_3_R function (Agrawal et al., 2010).

No evidence has been reported that the contribution of IP_3_R to SOCE in *Drosophila* occurs in mammalian neurons. However, in some mammalian cells, IP_3_Rs have been shown to co-localize with Orai1 (Lur et al., 2011) and interact with STIM1, Orai1, and TRPCs (Hong et al., 2011). The expression of IP_3_R isoform (IP_3_R_3_) was shown to be significantly lower in STIM1-deficient SH-SY5Y cells, meaning that STIM1 is a positive regulator of *ITPR3* gene expression in these cells (Pascual-Caro et al., 2020). IP_3_R_3_ is a Ca^2+^ channel that is localized mainly at the ER-mitochondrion junction, which transfers Ca^2+^ from the ER to mitochondria (Ivanova et al., 2014). Thus, STIM1 deficiency leads to a decrease in mitochondrial Ca^2+^ concentrations, leading to cell death. The overexpression of IP_3_R_3_ restores mitochondrial Ca^2+^ homeostasis and bioenergetics, ATP production, and cell survival in STIM1-KO neuronal-like cells (Pascual-Caro et al., 2020). These results provide evidence of a novel STIM1-IP_3_R_3_-mediated pathway of mitochondrial Ca^2+^ levels, the dysregulation of which contributes to neurodegeneration. Mitochondria from AD patients have lower Ca^2+^ uptake (Kumar et al., 1994), which is attributed to lower IP_3_R_3_ and STIM1 levels (Pascual-Caro et al., 2020).

### EB1 and EB3

The dynamic structure of dendritic spines is preserved mainly by actin filaments, and microtubules (MTs) are cytoskeleton-organizing components localized in dendrites and axons (Majewski and Kuznicki, 2015; Wu et al., 2017). Microtubules have been shown to enter dendritic spines and trigger spine head enlargement (Gu et al., 2008; Hu et al., 2008). This transport of MTs into dendritic spines appears to be involved in mechanisms of synaptic plasticity. Microtubule plus-ends contain end-binding (EB) proteins, which are divided into three types: EB1, EB2, and EB3 and have been shown to interact with STIM1 (Akhmanova and Steinmetz, 2010).

EB1/EB3-STIM1 complexes mediate ER movement in non-excitable cells (Grigoriev et al., 2008; Honnappa et al., 2009; Asanov et al., 2013). The STIM1-EB association sequesters STIM1 in MTs and prevents the excessive activation of SOCE (Chang et al., 2018). STIM1 regulates the dynamics of EB1/EB3, coupling the ER to MTs within filopodia and thus controlling growth cones in the nascent nervous system (Pavez et al., 2019). Additionally, recent research demonstrated that EB3 forms complexes with STIM2 that promote the formation of mushroom spines in hippocampal neurons, and the disintegration of these complexes results in the loss of mushroom spines (Pchitskaya et al., 2017). The overexpression of EB3 increases the proportion of mushroom spines and rescues their deficiency in hippocampal neurons in an AD mouse model. EB3 overexpression also rescues the loss of mushroom spines after STIM2 knockdown, whereas STIM2 overexpression does not restore mushroom spines after EB3 depletion. Neither STIM2 overexpression nor the activation of hippocampal TRPC6 increases spine neuronal SOCs or the proportion of mushroom spines in WT neurons. EB3 recruits various proteins to dendritic spines during synaptic plasticity, and STIM2 may be one of these cargo proteins (Pchitskaya et al., 2017). EB3 is involved in the regulation of dendritic spine morphology partly through its association with STIM2. Therefore, targeting EB3-STIM2 complexes may stabilize dendritic spines in AD patients (Pchitskaya et al., 2017).

### Synaptopodin

In cultured neurons, STIM1 interacts with anchoring proteins in the dendritic spine apparatus that consists of laminar smooth ER stacks. Synaptopodin (SP) is localized between ER stacks of the spine apparatus. This cytosolic actin and α-actinin-binding protein has been shown to be essential for the formation of this organelle (Deller et al., 2003). Synaptopodin is more common in spines with large-volume spine heads, where it regulates synaptic plasticity by controlling spine head enlargement during LTP in the CA1 region of the hippocampus and enhances glutamate-induced Ca^2+^ release in dendritic spines of cultured hippocampal neurons (Deller et al., 2003; Vlachos et al., 2009; Korkotian et al., 2014). Synaptopodin deficiency alleviated the AD symptoms in the 3xTg mice and restores normal synaptic plasticity (Aloni et al., 2019).

Synaptopodin was recently shown to regulate activity-dependent Ca^2+^ signaling by recruiting STIM1 to the post-synaptic density (PSD; Korkotian et al., 2014; Segal and Korkotian, 2014). In primary hippocampal neurons, SP co-localizes with STIM1 (Korkotian et al., 2014). The localization of STIM1 in spines depended on SP, in which this protein preferentially located STIM1 to mushroom spines, where this association was especially evident (Korkotian et al., 2014). These results indicate that SP interacts with STIM and Orai and thus may regulate the functionality of Ca^2+^ stores and determine synaptic plasticity.

As SP belongs to actin-binding proteins, it would be interesting to investigate whether STIM could also directly interact with actin in dendritic spines. It is worth mentioning the regulatory role of actin in spine morphogenesis and stabilization that is necessary for memory formation, the role in mechanisms related to synaptic plasticity and the contribution to AD pathology (Basu and Lamprecht, 2018; Pelucchi et al., 2020). Previous research has demonstrated that STIM1 may interact with actin, and actin remodeling was required to move STIM to the PM after store depletion in human platelets. Furthermore, the polymerization of actin filaments was necessary for association of STIM1 with TRPC1 (López et al., 2006). Similarly, actin fibers were shown to be involved in an alternatively spliced long variant of STIM1 oligomerization that precedes activation of Orai1 in myoblasts (Darbellay et al., 2011). Notably, Trebak's group reported that STIM1 controls formation of actin stress fibers, independently of Orai1 and Ca^2+^, thus thrombin-mediated disruption of endothelial barrier function (Shinde et al., 2013).

### Presenilins and CaMKII

Familial Alzheimer's disease (FAD) is caused by a dominant inherited mutation of presenilins (PSs; PS1 and PS2) and amyloid-β (Aβ) precursor protein (APP; Chakroborty and Stutzmann, 2014). Presenilins constitute catalytic components of the γ-secretase complex, which cleaves transmembrane APP to produce Aβ. PS1 mutations have been shown to change APP cleavage in favor of producing Aβ. This peptide accumulates, causing neuronal death in the cerebral cortex and hippocampal neurons, contributing to cognitive impairment and other pathological hallmarks of AD (Chakroborty and Stutzmann, 2014).

Endogenous PS1 and STIM1 have been shown to interact in human SH-SY5Y neuroblastoma cells and mouse primary cortical neurons (Tong et al., 2016). Tong et al. defined STIM1 as a new substrate of γ-secretase in a PS model of AD. The PS1-accociated γ-secretase complex cleaves the STIM1 transmembrane domain, reducing ORAI activation and diminishing SOCE (Tong et al., 2016). Dendritic spines in hippocampal neurons with mutant PS1 are destabilized, which is reversed by both a γ-secretase inhibitor and STIM1 overexpression. Although, the cleavage of STIM2 has not yet been established, its structural similarity to STIM1 suggests that it may also be a target of γ-secretase. Ryazantseva et al. reported the enhancement of SOCE in hippocampal neurons with a PS1 ΔE9 mutation (Ryazantseva et al., 2018). This PS1 mutation excludes 28 amino acids from the proteolytic cleavage site, resulting in the accumulation of uncleaved proteins. STIM1 accumulation results in its enhanced relocation to the PM, increasing the Orai1-TRPC association and enhancing SOCE (Ryazantseva et al., 2018). In turn, SOCE was attenuated directly in neurons from transgenic mice expressing human mutant PS1 A246E (Herms et al., 2003). The effect of PS1 on STIM1 appears to differ depending on the type of mutation. Greotti et al. reported that both PS1 and PS2 in SH-SY5Y cells reduce SOCE by reducing STIM1 expression levels (Greotti et al., 2019). However, this reduction does not depend on γ-secretase activity. Lower amounts of STIM1 protein were also found in FAD PS-expressing cells that were treated with a γ-secretase inhibitor. Moreover, chronic ER Ca^2+^ depletion or alterations of STIM1 expression levels did not affect SOCE under resting conditions (Greotti et al., 2019). These results may be considered an adaptive consequence of a prolonged reduction of ER Ca^2+^ levels.

Interestingly, Aβ itself decreases both STIM1 and STIM2 expression. Several studies have reported a link between Aβ-mediated STIM2 downregulation and the loss of synapses in animal models of AD (Bojarski et al., 2009; Fonseca et al., 2015; Popugaeva et al., 2015; Sanati et al., 2019). STIM2 protects mushroom spines from toxic effects of amyloid oligomers *in vitro* and *in vivo* in models of amyloid synaptotoxicity (Popugaeva et al., 2015). Sanati et al. reported that gold nanoparticles (AuNPs) reversed deteriorations of memory and spatial learning in Aβ-treated rat hippocampal neurons (Sanati et al., 2019). AuNPs delay the elongation of Aβ and dissociate existing Aβ to less toxic form, enhanced the expression of STIM protein, and potentiated the cAMP/PKA signaling cascade that modulates synaptic plasticity and influences learning and memory (Waltereit and Weller, 2003; Sanati et al., 2019). Additionally, AuNPs may directly increase cAMP levels and consequently recruit STIM2 and PKA to potentiate GluR1-dependent synaptic plasticity (Sanati et al., 2019).

In PS1 KI neurons, STIM2-mediated SOCE activates CaMKII and thus stabilizes mushroom spines (Sun et al., 2014). STIM2 is abundantly expressed in dendritic spines of hippocampal neurons, where it co-localizes with CaMKII. STIM2 overexpression rescues SOCE, restores CaMKII activity, and prevents dendritic spine loss. The conditional deletion of STIM2 reduced synaptic SOCE, thereby causing the loss of mushroom spines and eventually leading to the death of hippocampal neurons in mice that expressed FAD-associated PS1 variants (Sun et al., 2014). On the other hand, STIM2 was reported to inhibit I_CRAC_ and SOCE amplitude and enhance intracellular Ca^2+^ stores through PS1 M146V mutant expression in a cellular model of AD (Ryazantseva et al., 2013). Mushroom spine loss also occurred in an APP-KI mouse model of AD, which was reported to be attributable to the accumulation of Aβ in the medium of APP-KI neurons (Zhang et al., 2015). Aβ overactivates mGluR5, leading to higher ER Ca^2+^ levels, the downregulation of STIM2 expression, impairments in synaptic SOCE, and lower CaMKII activity (Sun et al., 2014; Popugaeva et al., 2015; Zhang et al., 2015). The pharmacological inhibition of mGluR5 or overexpression of STIM2 restores synaptic SOCE and prevents mushroom spine loss. Downregulation of the synaptic STIM2–SOCE–CaMKII pathway causes the loss of mushroom spines in both PS1-KI and APP-KI models of AD. Moreover, TRPC6 and Orai2 have been shown to form complexes with STIM2 in hippocampal dendritic spines (Zhang et al., 2016). Thus, TRPC6 activation, STIM2 overexpression, and SOCE positive modulators can rescue mushroom spine loss in hippocampal neurons from both PS-KI and APP-KI mouse models of AD (Sun et al., 2014; Zhang et al., 2016).

## Other STIM-Interacting Molecules

Recent studies have shown other regulators of STIM proteins, such as transcription factors and proteasome inhibitors that may negatively modulate their function. Here, we describe three such regulators.

### NEUROD2

Neurogenic differentiation factor 2 (NEUROD2) is one of the most important neurogenic transcription factors in the CNS (Guner et al., 2017), which mutation is associated with schizophrenia (Dennis et al., 2019). Contrary to previous research that showed that NEUROD2 is a transcriptional activator (Fong et al., 2012; Bayam et al., 2015), a recent study suggested that it may also limit *Stim1* expression in cortical neurons and consequently regulate Ca^2+^ influx in SOCE (Guner et al., 2017). Using a chromatin immunoprecipitation and sequencing approach in mouse postnatal cerebral cortical tissue, NEUROD2 was found to bind to an intronic element within the *Stim1* gene. The knockdown of *Neurod2* expression in cortical neurons increased STIM1 protein expression and resulted in the upregulation of SOCE, whereas its overexpression decreased SOCE. NEUROD2 activity is induced by Ca^2+^ influx via VGCCs, and depolarization-mediated Ca^2+^ influx via VGCCs appears to activate NEUROD2, which in turn fine-tunes the expression of STIM1 and SOCE and results in the STIM1-dependent inhibition of L-type VGCCs (Guner et al., 2017).

### Sp4

Sp4 is a transcription factor that regulates neuronal morphogenesis and function. Its stability depends on membrane potential. Sp4 level was increased in the brains of AD patients and reduced in the brains of bipolar disorder patients (Boutillier et al., 2007; Pinacho et al., 2011). A recent study reported that the maximal activation of SOCE under resting conditions promotes the degradation of Sp4 in cerebellar granule neurons *in vitro* (Lalonde et al., 2014). The lowering of extracellular K^+^ levels reduces neuronal excitability and stimulates the depletion of ER Ca^2+^ stores, resulting in STIM1 migration to the ER-PM junction and SOCE activation. SOCE inhibitors prevent the ubiquitination and degradation of Sp4 during low extracellular K^+^ levels. STIM1 knockdown also inhibits the degradation of Sp4, whereas a constitutively active STIM1 mutant (STIM1D76A) decreased Sp4 protein levels after depolarization (Lalonde et al., 2014). Neurons that were transfected with STIM1D76A were less likely to show immunopositive nuclei with Sp4 than WT STIM1. These findings suggest that STIM1 regulates Sp4 protein, meaning that Sp4 is a downstream effector of STIM1 and STIM1-mediated SOCE. We can assume that dysregulation of STIM1-mediated SOCE may induce abnormal Sp4 expression and promote the development of disorders such as bipolar disorder or AD.

### Proteasome Inhibitors

A recent study showed that sub-lethal doses of proteasome inhibitors, such as MG-132 and clasto-lactacystin-β-lactone (LA), decreased STIM1 and STIM2 levels in primary rat cortical neurons but did not affect either Orai1 or TRPC1 (Kuang et al., 2016). The loss of STIM1 and STIM2 proteins was also observed in SH-SY5Y neuroblastoma cells that had low levels of the proteasome subunit β type 5. Additionally, MG-132 and LA promoted autophagy and STIM1/STIM2 mobilization to lysosomes. Thus, inhibition of the ubiquitin-proteasome system, common in neurodegenerative disorders, may disrupt Ca^2+^ homeostasis by suppressing SOCE.

## Concluding Remarks

Calcium homeostasis in the CNS is vital for cell maintenance. As a second messenger, Ca^2+^ participates in plenty physiological processes and its level is regulated in a comprehensive way via the components localized in the PM (ion channels, exchangers, and pumps), as well as the components localized in the mitochondria, ER, Golgi apparatus, and nucleus. Under pathological conditions, Ca^2+^ homeostasis is dysregulated, with increased cytoplasm, mitochondrial, and changed ER Ca^2+^ concentration leading to apoptosis (Ureshino et al., 2019). Since the increasing evidence of the relevance of Ca^2+^ homeostasis in neuroprotection, we focused on the expression and function of Ca^2+^ signaling-related proteins, STIM partners and effectors, in terms of the effects on Ca^2+^ regulation and its potential use in the alleviation of the symptoms of neurodegenerative diseases.

Since the discovery of STIM and Orai proteins 15 years ago, they have been found to be the main components of SOCE but not the only components. Experimental evidence that was reviewed herein clearly demonstrates that STIM-Orai-mediated SOCE in the CNS is influenced by several regulators and STIMs have several effectors. We summarized the existing knowledge of target molecules of STIM proteins (Table 1 and Figure 3).

**Table 1:** STIM target molecules.

**Interacting protein**	**Identity of binding protein**	**Subcellular location**	**Function**	**STIM isoform**
Orai	Ca^2+^ channel	PM	Positive effector	STIM1 STIM2
TRP	Ca^2+^ channel	PM	Positive effector	STIM1
VGCC	Ca^2+^ channel	PM	Negative effector	STIM1
AMPAR	Ionotropic receptor	PM	Positive effector	STIM1 STIM2
NMDAR	Ionotropic receptor	PM	Negative effector	STIM1 STIM2
mGluR1	Metabotropic receptor	PM	Positive regulator	STIM1
septin	GTPase	PM lipids (cytoskeleton)	Positive/negative regulator	dSTIM
Homer	Scaffolding protein	PSD (cytoskeleton)	Negative regulator	STIM1
SP	Actin-binding protein	PSD (cytoskeleton)	Positive regulator	STIM1
CaMKII	Kinase	PSD (cytoskeleton)	Positive effector	STIM2
EB1, EB3	Microtubule-binding protein	Cytoplasm (cytoskeleton)	Positive effector/regulator	STIM1 STIM2
Golli proteins	Myelin basic protein	Cytoplasm	Positive regulator	STIM1
SARAF	Transmembrane regulatory factor	ER (predominant), PM	Negative regulator	STIM1
POST	Transmembrane protein	ER (predominant), PM	Positive regulator	STIM1
SERCA	Ca^2+^-ATPase	ER	Positive effector	STIM1
IP_3_R	Ca^2+^ channel	ER	Positive regulator/effector	dSTIM STIM1
PS1	Ca^2+^-leak channel, endoprotease	ER	Negative regulator	STIM1 STIM2
NEUROD2	Transcription factor	Nucleus	Negative regulator	STIM1
Sp4	Transcription factor	Nucleus	Negative effector	STIM1
MG-132/LA	Proteasome inhibitors	Cytoplasm/ nucleus	Negative regulator	STIM1 STIM2

*PM, plasma membrane; ER, endoplasmic reticulum; PSD, post-synaptic density*.

We can distinguish both positive (SEPT1, SEPT4, golli proteins, SP, POST, and EB) and negative (Homer, SARAF, SEPT7, PS1, NEUROD2, and proteasome inhibitors) regulators of STIM that affect STIM expression and structure and its movement to the PM or activation of Orai channels (Table 1). The majority of these regulators have an impact on STIM-Orai interactions, but both golli proteins and SARAF also influence STIM-TRPC associations. Interestingly, some of the regulators appear to function in two ways. SEPT1 and SEPT4 increase STIM–Orai-dependent SOCE, whereas SEPT7 inhibits STIM migration to ER-PM junctions and Orai activation. In turn, EB3 can restore the loss of mushroom spines after knocking down STIM2 (i.e., a regulator of STIM). Conversely, the dynamics of EB1/EB3 are regulated by STIM1 (i.e., an effector of STIM). Notably, in contrast to golli proteins in immune cells where it negatively regulates STIM-dependent SOCE, golli proteins in the brain appear to have a positive regulatory action on SOCE activity that is mediated by STIM1. We assume that the action of golli proteins differs depending on the type of cell tested. However, the exact molecular mechanisms that underlie the STIM1-golli protein interaction have not yet been defined.

The main effectors of STIM proteins are Orai and TRPC, which together constitute molecular components of SOCE. The evidence gathered in this review suggests that disturbances of the STIM-Orai- and STIM-TRPC-mediated SOCE pathway contribute to the pathogenesis of diverse neurodegenerative diseases. The STIM-Orai association is also vital for the regulation of neurogenesis in mammalian cells and production of proinflammatory cytokines in astroglia and murine microglia. In turn, the STIM-TRPC interaction is essential for the survival of DNs in animal models of PD. In addition to Orai and TRPC, STIM proteins can also control Ca^2+^ influx via other molecular channels, including L-type VGCCs, and receptors, such as AMPARs, NMDARs, mGluR1, and mGluR5. The interaction between STIM proteins and their molecular targets can both increase and decrease Ca^2+^ influx from the extracellular milieu to the cytoplasm. Associations between STIMs and Orai, TRPC, AMPARs, mGluR1, and mGluR5 elevate Ca^2+^ influx, whereas Ca^2+^ influx via L-type VGCCs and NMDARs is inhibited by STIMs. Interestingly, maximal SOCE activation occurs under resting conditions, whereas VGCC and NMDAR activation requires cell membrane depolarization. Depolarization activates Ca^2+^ influx via both NMDARs and L-type VGCCs and decreases Ca^2+^ content in the ER, thereby enhancing STIM1-mediated SOCE and decreasing Ca^2+^ influx via VGCCs and NMDARs. Thus, STIM proteins in neurons may regulate Ca^2+^ influx under both resting and action potential.

The relationship between STIM proteins and glutamate receptors is essential for different forms of synaptic plasticity. In primary rat hippocampal neurons, STIM2 promotes the phosphorylation and surface delivery of AMPARs, contributing to LTP. SOCE can be activated by synaptic NMDARs, thus also influencing LTP. Additionally, STIM1 was shown to control the plasticity of L-type VGCC-dependent dendritic spines in PNs and strengthen mGluR1-dependent synaptic transmission, thereby regulating cerebellar motor behavior. Other STIM protein effectors and regulators that reside outside the PM also contribute to synaptic plasticity. Synaptopodin recruits STIM1 to the PSD and regulates the function of Ca^2+^ stores and plasticity of spine heads during LTP. After cell depolarization, the STIM1-POST complex binds to SERCA and keeps it in close proximity to L-type VGCCs to promote ER Ca^2+^ replenishment during repetitive firing, regulating neuronal excitability and plasticity. The STIM1-POST complex appears to prevent excessive Ca^2+^ concentrations in the cytoplasm. In turn, the STIM1-IP_3_R_3_ interaction regulates basal Ca^2+^ levels in mitochondria. Interestingly, the dysregulation of both cytosolic and mitochondrial Ca^2+^ levels contributes to neurodegeneration in AD. In turn, the STIM2-EB3 and STIM2-CaMKII complexes promote the formation of mushroom spines and stabilize them. Targeting these complexes could be a novel way of stabilizing dendritic spines and thus improve memory in AD patients.

Studying STIM proteins and their partners in different subcellular compartments enables us to understand a wide range of processes that are regulated by these proteins. Future studies should examine the ways in which these regulators act in concert to modulate STIM activity during Ca^2+^ influx into the cell. The precise molecular mechanisms of action of STIMs together with these all regulators (e.g., in the activation/inactivation of Orai channels) also remain to be explored. Such studies will help to elucidate the pathological mechanisms that are involved in the development of various neurodegenerative diseases (e.g., AD, PD, and HD), affective disorders (schizophrenia, bipolar disorder), chronic pain, oxidative trauma, brain trauma, stroke, and epilepsy. Therefore, it appears that STIM proteins and their modulators/effectors may have potential therapeutic applications for the treatment of these diseases.

## Author Contributions

KS wrote and commented on the manuscript and prepared the figures. JG-B designed the manuscript, contributed to writing it, and prepared the final version. All authors contributed to the article and approved the submitted version.

## Conflict of Interest

The authors declare that the research was conducted in the absence of any commercial or financial relationships that could be construed as a potential conflict of interest.

## Abbreviations

AD: Alzheimer's disease
AMPAR: α-amino-3-hydroxy-5-methyl-4-isoxazolepropionic acid receptor
APP: amyloid-β precursor protein
Ca^2+^: calcium ion
CaMKII: Ca^2+^/calmodulin-dependent protein kinase II
CNS: central nervous system
CRAC: Ca^2+^ release-activated channel
DNs: dopaminergic neurons
dSTIM1: *Drosophila* STIM1
EB: end-binding protein
ER: endoplasmic reticulum
FAD: Familial Alzheimer's disease
HD: Huntington's disease
IP_3_: inositol-1,4,5-trisphosphate 3
IP_3_R: IP_3_ receptor
KI: knockin
KO: knockout
LTD: long-term depression
LTP: long-term potentiation
MBP: myelin basic protein
mGluR: metabotropic glutamate receptor
MT: microtubule
NEUROD2: Neurogenic differentiation factor 2
NMDAR: *N*-methyl-D-aspartate receptor
nNOS: neuronal nitric oxide synthase
NPC: neural progenitor cell
OASF: Orai1-activating small fragment
OPC: oligodendrocyte progenitor cell
PD: Parkinson's disease
PLA: proximity ligation assay
PLC: phospholipase C
PM: plasma membrane
PMCA: plasma membrane Ca^2+^ adenosine triphosphatase
PN: Purkinje neuron
POST: partner of STIM1
PS: presenilin
PSD: post-synaptic density
SARAF: SOCE-associated regulatory factor
SERCA: sarco/endoplasmic reticulum Ca^2+^-adenosine triphosphatase
siRNA: small-interfering RNA
SOAR: STIM–Orai-activating region
SOC: store-operated channel
SOCE: store-operated Ca^2+^ entry
SP: synaptopodin
STIM: stromal interaction molecule
TRPC: transient receptor potential cation channel subfamily C
TRPM: transient receptor potential cation channel subfamily M
VGCC: voltage-gated Ca^2+^ channel
WT: wildtype.
